# A multi-source data integration approach reveals novel associations between metabolites and renal outcomes in the German Chronic Kidney Disease study

**DOI:** 10.1038/s41598-019-50346-2

**Published:** 2019-09-27

**Authors:** Michael Altenbuchinger, Helena U. Zacharias, Stefan Solbrig, Andreas Schäfer, Mustafa Büyüközkan, Ulla T. Schultheiß, Fruzsina Kotsis, Anna Köttgen, Rainer Spang, Peter J. Oefner, Jan Krumsiek, Wolfram Gronwald

**Affiliations:** 1 Statistical Bioinformatics, Institute of Functional Genomics, University of Regensburg, Am Biopark 9, 93053 Regensburg, Germany; 2 Institute of Computational Biology, Helmholtz Zentrum München, Ingolstädter Landstraße 1, 85764 Neuherberg, Germany; 3 Department of Physics, University of Regensburg, Universitätsstraße 31, 93053 Regensburg, Germany; 4 Institute of Genetic Epidemiology, Department of Biometry, Epidemiology, and Medical Bioinformatics, Faculty of Medicine and Medical Center, University of Freiburg, 79106 Freiburg, Germany; 5 Department of Nephrology, Medical Center, University of Freiburg, 79106 Freiburg, Germany; 6 Institute for Functional Genomics, University of Regensburg, Am Biopark 9, 93053 Regensburg, Germany; 7 Institute of Computational Biomedicine, Weill Cornell University, New York, NY 10021 USA

**Keywords:** Data integration, Machine learning, Statistical methods

## Abstract

Omics data facilitate the gain of novel insights into the pathophysiology of diseases and, consequently, their diagnosis, treatment, and prevention. To this end, omics data are integrated with other data types, e.g., clinical, phenotypic, and demographic parameters of categorical or continuous nature. We exemplify this data integration issue for a chronic kidney disease (CKD) study, comprising complex clinical, demographic, and one-dimensional ^1^H nuclear magnetic resonance metabolic variables. Routine analysis screens for associations of single metabolic features with clinical parameters while accounting for confounders typically chosen by expert knowledge. This knowledge can be incomplete or unavailable. We introduce a framework for data integration that intrinsically adjusts for confounding variables. We give its mathematical and algorithmic foundation, provide a state-of-the-art implementation, and evaluate its performance by sanity checks and predictive performance assessment on independent test data. Particularly, we show that discovered associations remain significant after variable adjustment based on expert knowledge. In contrast, we illustrate that associations discovered in routine univariate screening approaches can be biased by incorrect or incomplete expert knowledge. Our data integration approach reveals important associations between CKD comorbidities and metabolites, including novel associations of the plasma metabolite trimethylamine-N-oxide with cardiac arrhythmia and infarction in CKD stage 3 patients.

## Introduction

The advent of new omics technologies, offering high coverage at an affordable price, has changed the landscape of large-scale clinical studies. More and more population-based trials collect not only information about phenotypes, traditional clinical parameters, and demographic variables, but also extensive omics data, e.g. KORA^1,2^, and TwinsUK^3^. This typically results in a large set of complex patient data, which needs to be statistically analyzed.

One example of such a large-scale, population-based study is the German Chronic Kidney Disease (GCKD) study, comprising about 5217 chronic kidney disease (CKD) patients. CKD constitutes one of the largest burdens on the world’s health system^4^, with about 200 cases per million residents per year in many countries^5^, and a global occurrence of 50% in high-risk subpopulations^6^. As it progresses, CKD leads to a large number of adverse clinical symptoms, and in some cases may progress to complete renal failure, termed end-stage renal disease (ESRD)^7^. It is linked to elevated all-cause and cardiovascular mortality, acute kidney injury, cognitive decline, anemia, mineral and bone disorders, and fractures^4,7,8^. Moreover, it is often accompanied by numerous other systemic diseases, such as cardiovascular disease, hypertension, obesity, and diabetes^6,9,10^.

The GCKD study was designed to improve our understanding of the causes, course, and risk factors of progressive loss of renal function^11^. To that end, demographic, phenotypic, and clinical parameters of 5217 CKD patients were assessed^11,12^. Additionally, NMR metabolic fingerprints of plasma specimens collected at the study baseline were acquired. Metabolic markers are expected to better reflect the current state of the kidney than, e.g., genomic, transcriptomic, or proteomic markers^13^. In total, approximately 900 parameters of diverse data sources, which potentially have complex dependencies amongst each other, have been assembled. Here, we aim at the identification of novel and the confirmation of known relationships between these complex layers of information. Particularly, our goal is the detection of metabolic features related to CKD and its diverse comorbidities.

Data integration tries to uncover such relationships. However, it presents both a conceptual and a practical challenge. Conceptually, we are faced with complex, often synergistic effects between variables within and across different layers of information, where the variables are potentially of different data types. Practically, we have to deal with a large-scale dataset covering close to a thousand variables and several thousand measurements, leaving the researcher with a lot of possible hypotheses that require extensive validation. The practical and conceptual issues are inseparably connected. Assuming more complex and realistic models requires more parameters and their estimation becomes computationally more and more challenging.

Numerous methods have been proposed to analyze complex datasets, ranging from metabolome-wide association studies and multivariate statistical analysis to data-driven network-based approaches^14,15^. Metabolome-wide association studies are applied routinely to identify associations between metabolites and phenotypes, but they inherently ignore complex, multivariate relationships between variables. Approaches based on probabilistic graphical models can reveal complex relationships, but they are usually limited to one specific data type, e.g., Gaussian Graphical Models (GGMs) for continuous (Gaussian) variables or discrete Markov Random Fields (dMRF) for categorical variables^16^. More recently, probabilistic graphical models have been extended to include different data types simultaneously^16,17^, although such methods have not yet entered biomedical research.

Univariate screening approaches ignore effects of confounding variables. These can be either incorporated by correcting the data or by adapting the statistical test. Formally, this corresponds to estimating the partial correlation coefficient ${\rho }_{XY\cdot Z}$, which denotes the partial correlation between *X* and *Y* given the confounders *Z*. If ${\rho }_{XY\cdot Z}\ne 0$, then there is an association between *X* and *Y* given *Z*, where the size and sign of ${\rho }_{XY\cdot Z}$ reflects the strength and sign of an association, respectively. An inherent problem in the estimation of ${\rho }_{XY\cdot Z}$ is that it is not *a priori* clear which variables to include in *Z*. To solve this issue, we need to estimate the joint probability of *X*, *Y*, and *Z*.

Here, we estimate the joint probability of comprehensive patient data assembled within the GCKD study. The included variables are either continuous or categorical. Thus, this analysis requires the estimation of a so-called Mixed Graphical Model (MGM)^16,17^. We first show how this integrative analysis reveals known relationships between variables. Second, we illustrate that the discovery of associations in univariate screening approaches is biased by incorrect or incomplete expert knowledge. Third, we demonstrate that our data integration approach overcomes this issue. Finally we give an example, where novel associations of the plasma metabolite trimethylamine-N-oxide (TMAO) with cardiac arrhythmia and infarction are revealed. Here, we show that the discovered associations remain significant after variable adjustment based on expert knowledge. Throughout the article, we substantiate our findings by evaluating the predictive performance of the estimated model on validation data.

## Results

### Mixed graphical models

Our data integration approach is based on Mixed Graphical Models (MGMs). MGMs are undirected probabilistic graphical models, where the conditional dependencies between different variables, the so-called nodes or vertices, are represented as edges in a network. Thus, two nodes are connected by an edge only if their association or interaction cannot be explained by any other node in the graphical model, or equivalently, if their partial correlation coefficient is unequal zero. Consequently, probabilistic graphical models eliminate spurious associations between variables and can potentially reveal new associations adjusted for all other variables in the dataset.

### Algorithmic implementation

For the current dataset, which included 879 variables and 3705 patients (measurements), we had to estimate, in total, 388521 edge weights.

Therefore, we implemented an efficient algorithm to estimate MGMs as described in the Supplementary Methods section 1.3. To this end, we used the pseudo-log-likelihood method of Lee and Hastie^17^ and implemented a proximal algorithm to include a LASSO penalty on the edge weights^18^. Furthermore, our implementation uses Nesterov’s acceleration and adaptive restarts^19,20^. It is available in the Supplementary Material File 2.

### Data integration workflow

**Figure 1:**
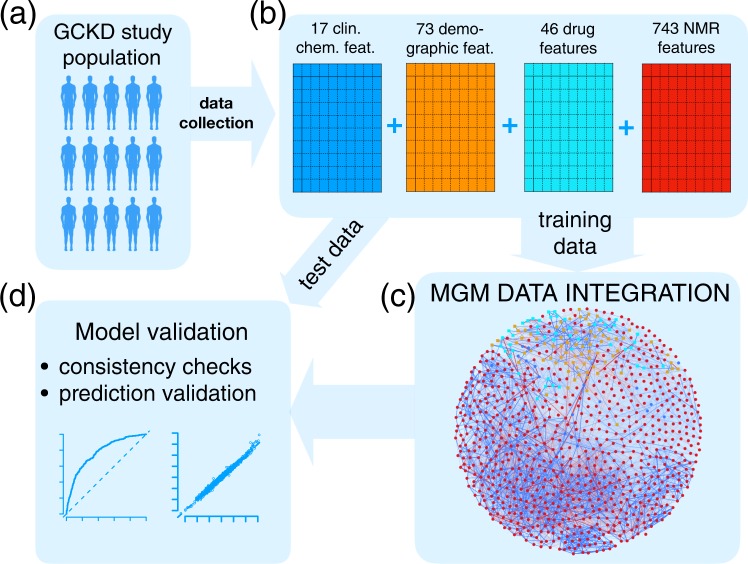
Scheme of the Mixed Graphical Model (MGM) data integration approach. (**a**) Of the data ascertained from the GCKD study population, (**b**) a total of 17 clinical chemistry parameters (blue), 73 demographic parameters (orange), 46 drug treatment parameters (cyan), and 743 NMR spectral features (red) were chosen. The complete dataset was split into a training and a test cohort, respectively. The first (**c**) was used to estimate an MGM, modeling all conditional dependencies between all variables, whereas the latter (**d**) was used for MGM model validation. In the network representation of the estimated MGM, blue nodes represent clinical chemistry parameters, orange nodes represent demographic variables, cyan nodes represent drug treatment information, and red nodes correspond to NMR buckets. Continuous variables are represented as circles and discrete variables as rectangles. Positive and negative associations are shown as blue and red edges, respectively. The strength of the association, i.e., the weight of the corresponding coefficient, is encoded by the edge width.

A schematic illustration of our data integration workflow is shown in Fig. 1. Starting point is the GCKD study, Fig. 1a. Here, we included a total of 3705 GCKD study participants. Since our data integration approach requires a complete data matrix, all study participants, for whom at least one demographic, clinical, and/or metabolic data point was missing, were excluded prior to analysis. We split the complete data set into a training set of 2470 study participants, corresponding to 2/3 of the complete cohort, and a test set of 1235 study participants, comprising the remaining study participants, as illustrated in Fig. 1b. For the current study, we included 17 clinical chemistry parameters, 73 demographic parameters, and 46 different drug treatments, all assessed at the study baseline. Each of the three different information layers is represented by a blue, orange, and cyan schematic data matrix in Fig. 1b, respectively. For each study participant, a one-dimensional (1D) ^1^H nuclear magnetic resonance (NMR) spectrum of a baseline EDTA plasma specimen was acquired. In total, 743 metabolic features were extracted from each spectrum, as described in the Supplementary Methods section 1.2. This information layer is represented by the red data matrix in Fig. 1b. In summary, the complete variable space included 879 variables, with 768 continuous and 111 discrete variables, respectively.

Next, the MGM was estimated on the training data, Fig. 1c, and validated on independent test data, Fig. 1d. Here and throughout the article, clinical chemistry parameters are represented as blue, demographic variables as orange, drug treatment information as cyan, and NMR buckets as red nodes, respectively. Positive and negative associations are shown as blue and red edges, respectively, where the edge width encodes the absolute edge strength. Continuous variables are shown as circles and discrete variables as rectangles.

### MGM data integration reveals known associations and combines them to reliable prediction models

First, we will present several sanity checks for our MGM approach. We will illustrate how the MGM reveals known associations in the context of a renal performance marker, for two frequent comorbidities, and for a lifestyle factor.

#### Glomerular filtration rate (GFR)

The glomerular filtration rate (GFR) is one of the most important markers of renal function routinely assessed in CKD patients. It defines the fluid volume filtered by the glomeruli per unit time. Several formulas are available to estimate the GFR. Here, we included estimated GFR (*eGFR*) values calculated according to the CKD-EPI equation^21^.

**Figure 2:**
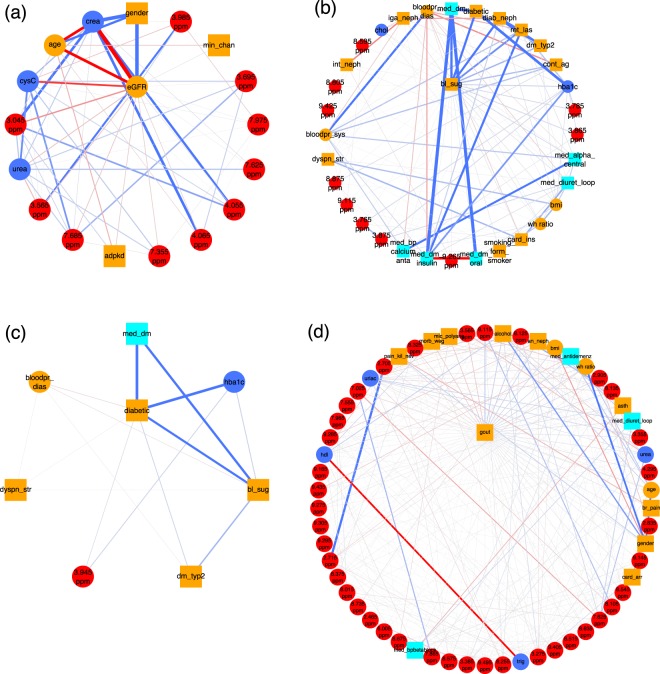
(**a**) First order neighborhood of CKD-EPI eGFR values based on serum creatinine (*eGFR*). The first order neighborhood of a node, e.g., *eGFR*, includes, next to the node of interest, all nodes in the estimated MGM, which are directly connected to this particular node by only one edge. These are the only nodes which have been identified as being directly associated with *eGFR*. Positive associations are represented as blue, negative associations as red edges, respectively. The strength of the estimated association is encoded by the edge width. The edges are ordered according to their strength in a clock-wise manner for positive, and in an anti-clock-wise manner for negative associations, respectively. *eGFR* is strongly negatively associated with serum creatinine (*crea*) (edge weight = −11.19), strongly positively associated with male gender (*gender*) (edge weight = 7.51), and negatively associated with age (*age*) (edge weight = −2.71). Negative associations are revealed between *eGFR* and serum cystatin C values (*CysC*) (edge weight = −0.76), and the NMR bucket at 3.045 ppm (edge weight = −0.37), corresponding to creatinine, respectively. (**b**) First order neighborhood of elevated blood sugar (*bl_sug*) and (**c**) classification as diabetic patient (*diabetic*). Strong associations can be observed between *bl_sug* and diabetes medications (*med*_*dm*) (edge weight = 1.52), *diabetic* (edge weight = 1.27), and diabetic nephropathy (*diab*_*neph*) (edge weight = 1.15), respectively. Other strong associations are present between *diabetic* and *med_dm* (edge weight = 2.89), and the HbA1c value (*hba*1*c*) (edge weight = 2.37), respectively, as well as between 2 NMR buckets at 3.785 ppm (edge weight = 0.14) and 3.865 ppm (edge weight = 0.13), both corresponding to D-glucose, and *bl_sug*, and between *diab*_*neph* and classification as type-2 DM patient (*dm_typ2*) (edge weight = 7.1) and retinal laser therapy due to diabetes (*ret*_*las*) (edge weight = 0.47), respectively. (**d**) First order neighborhood of gout (*gout*). Strong positive associations between this phenotype and the NMR bucket at 8.115 ppm (edge weight = 0.27), corresponding to unidentified small peptides, alcohol (edge weight = 0.20), 8.125 ppm (edge weight = 0.19) (unidentified small peptides), as well as analgetic nephropathy (*an_neph*) (edge weight = 0.17), and strong negative associations with the NMR bucket at 3.565 ppm (edge weight = −0.16), identified as glycine, can be observed. Gout is also connected to bmi (edge weight = 0.15), anti-dementia medication (*med_antidemenz*) (edge weight = 0.14), waist-hip ratio (*wh ratio*) (edge weight = 0.13), and Morbus Wegener (*morb_weg*) (edge weight = −0.13). Supplementary Table S1 lists all abbreviations for the clinical parameters.

CKD-EPI takes as input serum creatinine (*crea*), age, gender, and race. In Fig. 2a, we show the first order neighborhood of *eGFR*. In general, the first order neighborhood of a node, here *eGFR*, comprises all nodes in the MGM, which are directly connected to this particular node by only one edge, as well as the node of interest itself. These are the only nodes which have been identified as being directly associated with *eGFR*. The algorithm correctly identified age, gender, and serum creatinine in the first order neighborhood, which are used for the computation of *eGFR*. Race was not included as a variable, as all GCKD study participants were Caucasian, and, thus, the respective connection could not be observed.

In addition, we observed associations of eGFR with cystatin C (*cysC*), and NMR buckets assigned to creatinine (3.045 ppm, 4.055 ppm, and 4.065 ppm). Serum cystatin C, besides serum creatinine, is used as an endogenous marker to estimate GFR, e.g., in the CKD-EPI equation based on cystatin C^22^, which explains the found association between cystatin C and eGFR.

**Figure 3:**
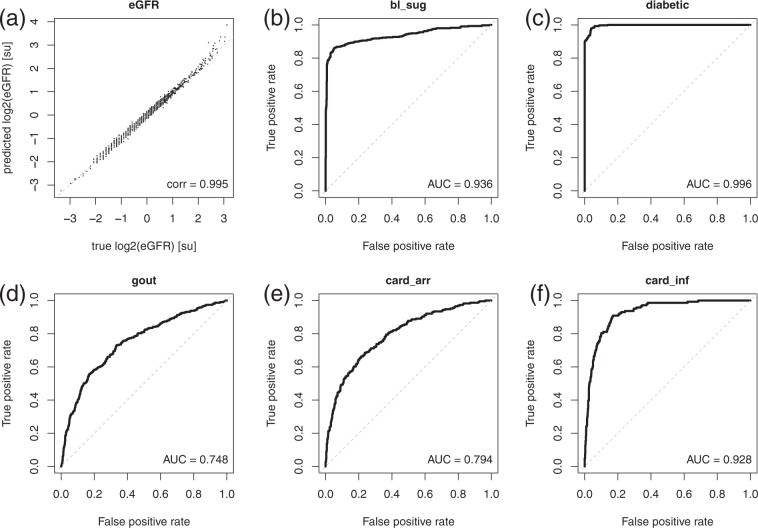
(**a**) The diagram shows the predictions of *eGFR* on the *y*-axis (in standard units [su]) based on the neighbors of *eGFR* on independent test data compared to the true values plotted on the *x*-axis. Predictions agree almost perfectly with the true values as indicated by the correlation coefficients *corr* between true and predicted values given in the lower right corners. The receiver operating characteristic (ROC) curve for predicting elevated blood sugar based on its neighborhood is shown in (**b**). The *x*-axis here represents the false, whereas the *y*-axis represents the true positive rate, respectively. The dashed line gives the diagonal, corresponding to the predictive performance of a randomly generated model. In the lower right corner, the area under the ROC curve (AUC), an indicator of the predictive power of a classifier, is given. A perfect classifier would achieve an AUC of 1 on independent test data, whereas a randomly generated classifier with no predictive power would achieve an AUC of 0.5, respectively. (**c**–**f**) Show the ROC curves for the neighborhood models of the medical diagnosis of a patient as being diabetic, gout, cardiac arrhythmia (*card*_*arr*), and cardiac infarction (*card*_*inf*), respectively.

In a second step, we validated the predictive performance of the detected neighborhood. For this purpose, we used the respective edge weights as a linear signature that predicts *eGFR* on test data, as can be seen in Fig. 3a. We observe that values agree with a correlation coefficient of *cor* = 0.995.

The corresponding analysis for a second renal performance marker, the urinary albumin-to-creatinine ratio, can be found in the Supplementary Results section 2.1.

#### Diabetes

One of the most common comorbidities of CKD is type-2 diabetes mellitus (T2DM). Untreated T2DM is characterized by high blood glucose. In the GCKD study, elevated blood sugar is included as a discrete variable (*bl_sug*).

The MGM correctly identifies NMR buckets corresponding to D-glucose signals, namely 3.785 ppm, 3.865 ppm, 3.875 ppm, and 3.765 ppm, in the first-order neighborhood of elevated blood sugar, Fig. 2b. The three strongest connections are between elevated blood sugar and diabetes medications (*med_dm*), the classification as diabetes patient (*diabetic*), and diabetic nephropathy (*diab_neph*), respectively.

In Fig. 2c, we show the first-order neighborhood of *diabetic*, which exhibits a strong connection to diabetes medications (*med_dm*) and glycated hemoglobin (HbA1c) (*hba1c*). This reflects the definition of a diabetic patient within this study: a patient is defined as diabetic, if he had a pHbA1c ratio ≥6.5% or took at least one medication with an active component classified as “A10”, i.e. insulin and other diabetes medications. The pHbA1c ratio reflects the two to three month average plasma glucose level^23^ and is here associated with elevated blood sugar independently of diagnosed diabetes.

Other strong connections were observed between blood sugar and retinal laser therapy due to diabetes (*ret_las*), as well as classification as T2DM patient (*dm_typ2*). All three variables are positively connected to diabetic nephropathy (*diab_neph*), as a study participant is stated to suffer from diabetic nephropathy, if he/she suffers from type-1 or type-2 DM or from other diabetic nephropathies.

The corresponding neighborhoods of *bl_sug* and *diabetic* are highly predictive. The areas under the receiver operating characteristic (AUC-ROC) curves are 0.936 and 0.996 on the test data (Fig. 3b,c, respectively).

#### Gout

Gout is a common comorbidity in CKD patients^24,25^. The first order neighborhood of gout (*gout*) as shown in Fig. 2d comprises a number of clinical and metabolic variables. This phenotype exhibits strong positive associations with the NMR buckets at 8.115 ppm and 8.125 ppm corresponding to unidentified small peptides. An NMR bucket at 3.565 ppm, which could be assigned to glycine, is strongly negatively associated with gout.

Glycine has been reported to negatively correlate with gout in other metabolic studies^26^. It is involved in purine metabolism and is a precursor of uric acid^26^. Decreased plasma levels of glycine in patients with gout might be caused by the increased production of uric acid in these patients^26^. Note that uric acid has also been positively associated with gout in our study.

Interesting associations between gout and clinical variables are present for high alcohol consumption (positive association), analgetic nephropathy (*an*_*neph*) (positive association), bmi (positive association), microscopic polyangiitis (*mic*_*polyang*) (negative association), anti-dementia medication (*med*_*antidemenz*) (positive association), waist-hip ratio (*wh ratio*) (positive association), and Morbus Wegener (negative association). Alcohol consumption, age (here positively associated with gout), male gender (here weakly positively associated with gout), medications such as loop diuretics (here positively associated), and obesity are known risk factors of gout^27,28^.

The first order neighborhood of gout is predictive for gout on independent test data with an AUC of 0.748 (Fig. 3d). In the Supplementary Results section 2.2 the first order neighborhood of high alcohol consumption is described. Interestingly, the strongest association was observed between high alcohol consumption and male gender.

### Associations in univariate screening approaches can be biased by incorrect or incomplete expert knowledge, while MGMs intrinsically account for confounding variables

Here, we compare two different approaches to screen for associations, i.e. a standard univariate regression and the MGM data integration approach. We will illustrate that the associations discovered by the MGM are more robust for *post hoc* confounder adjustment than univariately screened associations.

#### Univariate screening

**Figure 4:**
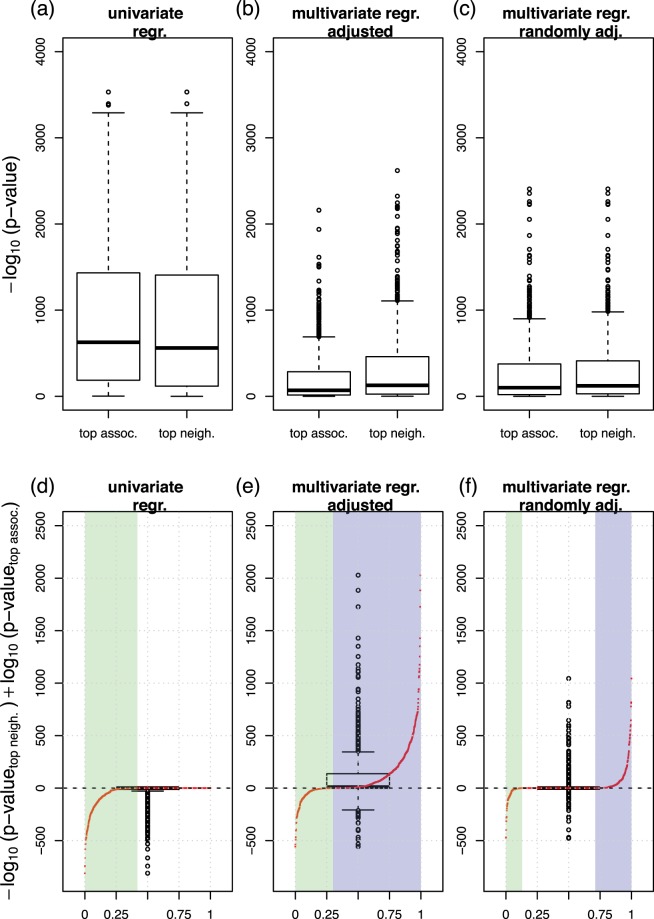
Effects of variable adjustment on univariate or MGM association analysis in the training set. (**a**) “top assoc.” shows the distribution of −log_10_(*p*-values) derived from a univariate regression. Here, we calculated *p*-values between all possible pairs of variables and collected all top associations. “top neigh.” shows the analogous distribution, where the top feature was selected by largest absolute edge weight in the MGM neighborhood. (**b**) The corresponding plot, where the *p*-values were corrected by the top five confounder variables of the univariate and MGM screening, respectively. (**c**) The corresponding plot, where we adjusted for the same five randomly selected features for both methods. (**d**–**f**) Show the differences between “top neigh.” and “top assoc.” in (**a**) to (**c**), respectively: (**d**) shows the −log_10_(*p*-values) of the MGM approach minus those of the univariate screening in (**a)**, **(e**) shows the corresponding plot after adjusting for the respective top confounders, as shown in (**b**), and (**f**) shows the corresponding plot after adjusting for the randomly selected confounders, as shown in (**c**). The red points in each figure contrast the values on the *y*-axis with their respective rank. On the *x*-axis, the highest positive difference corresponds to 1 and the most negative to 0. The green shaded areas correspond to rank percentiles of negative, the violet shaded areas correspond to rank percentiles of positive differences, respectively.

We first screened for univariate associations between variables by regressing each variable on every single remaining variable in the dataset. We performed linear or logistic univariate regression analysis, depending on whether the response variable followed a Gaussian or a binomial distribution, respectively. For each response variable, we ranked the univariate predictor variables according to their association strength in terms of significance, here −log_10_(*p*-values). The predictor with the largest −log_10_(*p*-value) was considered as the “top association”. Figure 4a column “top assoc.” shows the distribution of −log_10_(*p*-values) of all top associations.

**Figure 5:**
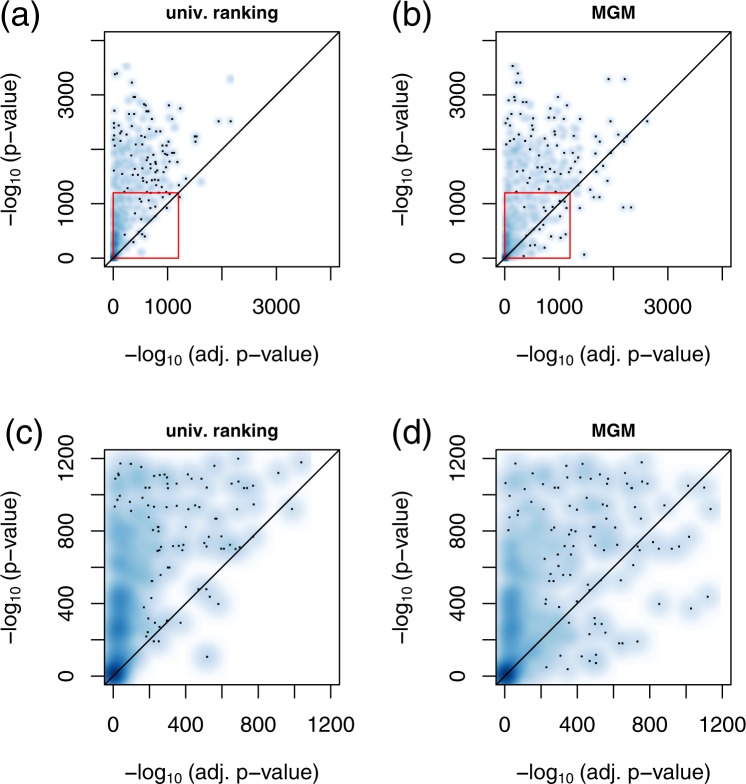
Smooth scatter plot of adjusted *p*-values (*x*-axis) versus unadjusted (univariate) *p*-values (*y*-axis) for the univariate screening (**a**), and the MGM (**b**) in the training set. The red rectangles mark excerpts shown in detail in (**c**,**d**), respectively.

Next, we repeated the regression analysis, but now in a multivariate fashion, simulating a regression analysis with confounder adjustment. We regressed each variable on its “top associated” variable and on the next five most significant predictors simultaneously, and recorded the −log_10_(*p*-value) of the “top associated” variable. The corresponding distribution is shown in Fig. 4b “top assoc”. We observe that the associations appear substantially weaker after variable adjustment. This trend is also visible from Fig. 5a, where we contrast adjusted (*x*-axis) with unadjusted (*y*-axis) −log_10_(*p*-values). Here, we obtained almost exclusively values in the upper half plane, see also Fig. 5c. Finally, we regressed each response variable on its “top associated” predictor and on five additional variables randomly drawn out of the next top 10 univariate predictors. The corresponding distribution can be found in Fig. 4c “top assoc”. Again, −log_10_(*p*-values) of the top associations in this multivariate regression scenario appear much weaker than in the univariate screening of Fig. 4a.

#### Associations in MGMs are intrinsically corrected for confounding variables

We performed an analogous analysis for our MGM approach, but now, we ranked the predictors of each response variable according to their edge weights (from largest to lowest absolute value). The predictor with the largest absolute coefficient was considered as the “top neighbor”. In a *post-hoc* analysis, we regressed each response variable on its top MGM neighbor, and show the distribution of corresponding −log_10_(*p*-values) in Fig. 4a, column “top neighbor”. Figure 4b column “top neighbor” shows −log_10_(*p*-values) for these top MGM neighbors, but now adjusted for the next top 5 neighbors in the respective MGM neighborhood. We observe that the significance decreases, as previously for the univariate screening, albeit, this effect is less pronounced. If we adjust for the same randomly drawn features as for the univariate approach, we obtain the results in Fig. 4c, which show the same trend. For Fig. 4d–f, we subtracted the left values (“top assoc.”) from the right values (“top neighbor”) in Fig. 4a–c, respectively. In addition, we contrast those differences with their respective rank as red points. The *x*-axis now corresponds to the rank percentiles, starting with the most negative difference at 0, and the highest positive difference at 1, respectively.

In Fig. 4d, all differences are at or below zero. Approx. 42% of the difference values are negative (see green highlighted area), and the remaining differences are zero. After confounder adjustment, Fig. 4e, the differences are predominantly positive. Considering the rank distribution, 70% of the difference values are positive (highlighted in violet), and only 30% are negative (highlighted in green). This indicates, on average, a higher significance of the top features selected by the MGM after variable adjustment. Figure 4f shows the corresponding results after an adjustment for the same randomly selected features for “top assoc.” and “top neighbor”. Now, 30% of the differences are positive, only 12% are negative, and most of them are equal to zero.

#### MGMs can reveal associations which remain hidden or underestimated in univariate approaches

In Fig. 5b we contrast adjusted with unadjusted values of −log_10_(*p*-values) for the MGM approach. We observe that several variables are stronger associated with each other after variable adjustment compared to the unadjusted scenario. This is in contrast to the univariate approach, Fig. 5a, where almost all values were located in the upper half plane. A comparison between unadjusted and randomly adjusted −log_10_(*p*-values) for the univariate and the MGM approach can be found in Supplementary Fig. 3.

An interesting example, how an association is revealed in a multivariate context, can be observed in the context of *gout*. One of the most important associations in the neighborhood of *gout* is *alcohol* (Fig. 2d), which was only ranked at position 216 in the univariate screening. Gender, in contrast, whose association is on rank 6 in a univariate screening, appears only at position 26 in the MGM. As can be clearly seen from Supplementary Fig. 2 male gender is the top neighbor of high alcohol consumption. Thus, the strong link between gout and male gender that was observed in univariate screening appears to be an indirect association mediated through alcohol consumption.

Plots corresponding to Figs 4 and 5 for the validation data are shown in Supplementary Figs 4–6 and strongly support our observations.

### TMAO is associated with cardiac infarction and cardiac arrhythmia

**Figure 6:**
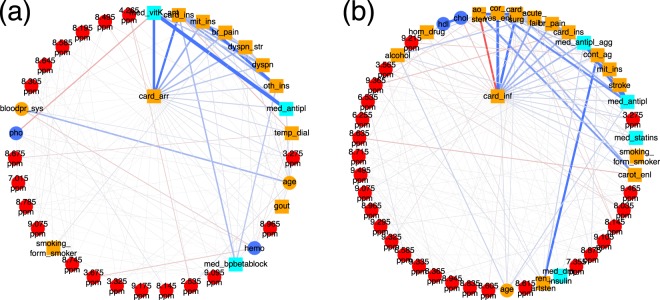
First order neighborhood of (**a**) cardiac arrhythmia (*card*_*arr*) and (**b**) cardiac infarction (*card*_*inf*). *card*_*arr* is strongly connected to vitamin K antagonists (*med*_*vitK*_*ant*) (edge weight = 1.50), heart failure (*card_ins*) (edge weight = 1.0), mitral valve insufficiency (*mit_ins*) (edge weight = 0.52), angina pectoris (*br_pain*) (edge weight = 0.39), dyspnea during physical strain (*dyspn*_*str*) (edge weight = 0.38) and during the night (*dyspn*) (edge weight = 0.26), other heart valve anomalies (*oth*_*ins*) (edge weight = 0.18), anti thrombotic drugs (*med*_*antipl*) (edge weight = 0.17), temporary dialysis (*temp_dial*), and were positively associated with an NMR bucket at 3.275 ppm (edge weight = 0.12), identified as trimethylamine-N-oxide (TMAO) and minor signals of D-glucose and betaine. *card*_*inf* is strongly connected to coronary angiopathy (*cor*_*ves*_*enl*) (edge weight = 1.80), cardiac surgery (*card*_*surg*) (edge weight = 1.32), aortic valve stenosis (*ao*_*sten*) (edge weight = −0.65), acute renal failure (*acute_fail*) (edge weight = 0.55), angina pectoris (*br*_*pain*) (edge weight = 0.49), heart failure (*card*_*ins*) (edge weight = 0.43), antiplatelet therapy (*med*_*antipl*_*agg*) (edge weight = 0.41), catheter angiography of peripheral arteries including angioplasty of a peripheral artery (*cont*_*ag*) (edge weight = 0.21), mitral valve insufficiency (*mit*_*ins*) (edge weight = 0.20), stroke (*stroke*) (edge weight = 0.19), serum cholesterol levels (*chol*) (edge weight = −0.16), anti thrombotic drugs (*med_antipl*) (edge weight = 0.16), and an NMR bucket at 3.275 ppm (edge weight = 0.14).

Cardiovascular diseases and complications are typical comorbidities and outcomes in patients with CKD. Here, we focus on the first order neighborhood of two common phenotypes, i.e., cardiac arrhythmia (*card_arr*) and cardiac infarction (*card_inf*), shown in Fig. 6a,b, respectively. Both variables are associated with a number of demographic and drug information parameters.

For patients diagnosed with cardiac arrhythmia (Fig. 6a) associations with vitamin K antagonists (*med*_*vitK*_*ant*) (positively associated), heart failure (*card_ins*) (positively associated), mitral valve insufficiency (*mit_ins*) (positively associated), angina pectoris (*br_pain*) (positively associated), dyspnea during physical strain (*dyspn*_*str*) and during the night (*dyspn*) (both positively associated), diagnosis of other heart valve anomalies (*oth*_*ins*) (positively associated), and antithrombotic drugs (*med*_*antipl*) (positively associated) were observed.

Patients who suffered a cardiac infarction (Fig. 6b) were likely (positively associated) to have received a diagnosis of coronary angiopathy (*cor_ves_enl*), to have undergone cardiac surgery (*card_surg*), were less likely to have been diagnosed with aortic valve stenosis (*ao*_*sten*) (negatively associated), suffered from CKD due to acute renal failure events (*acute_fail*) (positively associated), were likely to have experienced angina pectoris (*br*_*pain*) and heart failure (*card_ins*), and to have received antiplatelet therapy (*med*_*antipl*_*agg*). They were also likely to have underwent a catheter angiography of peripheral arteries including an angioplasty of a peripheral artery (*cont*_*ag*), to suffer from mitral valve insufficiency (*mit*_*ins*), or a stroke (*stroke*), and they also had, on average, lower serum cholesterol levels (*chol*) (negatively associated), probably since they received more statins.

Most interestingly, both cardiac infarction and cardiac arrhythmia were positively associated with an NMR bucket at 3.275 ppm, which could be identified as trimethylamine-N-oxide (TMAO) and minor signals from D-glucose and betaine. In the case of cardiac arrhythmia, this NMR bucket was ranked on position 10 in the MGM approach, whereas in the univariate screening approach, it was located on rank 13. In the univariate screening approach for variables associated with cardiac infarction, the NMR bucket at 3.275 ppm was ranked on position 17, whereas, in the MGM approach, it was located on position 13.

Increased plasma levels of TMAO have just recently been described as a marker for atrial fibrillation independent of hypertension, BMI, smoking, diabetes, or intake of total choline^29^, and have been associated with a higher incidence of major adverse cardiovascular events (death, myocardial infarction, or stroke)^30^.

Supplementary Fig. 7 displays the distribution of NMR signal intensities at 3.275 ppm for both patients without and with cardiac arrhythmia for training and test data, respectively. Comparing both distributions by a Student’s *t*-test yields a *p*-value of 5.3 · 10^−10^ and 6.8 · 10^−6^ for the training and test cohort, respectively. To explore possible confounding effects, we adjusted the NMR bucket intensities of 3.275 ppm for hypertension, BMI, smoking, and diabetes, which have been reported as confounders^29^. Intake of total choline was not accessed in the GCKD study and could therefore not be used for confounder adjustment. The corresponding boxplots for the residuals of NMR bucket intensities at 3.275 ppm are shown in Supplementary Fig. 7b,d, respectively. The respective *p*-values are still significant with values of 2.3 · 10^−7^ and 0.012 for training and test set, respectively.

The first order neighborhoods of cardiac arrhythmia and cardiac infarction are highly predictive on independent test data with AUCs of 0.80 and 0.93, respectively (Fig. 3f,g).

## Discussion

We have shown that MGMs are a versatile tool for the integration of both continuous (Gaussian) and categorical variables. Here, we used data collected from patients suffering from CKD and included variables comprising clinical and demographic parameters, medications, and blood plasma metabolites assessed by NMR spectroscopy. We could reveal several known relationships, such as the definitions of UACR, eGFR, and diabetes. More interestingly, we observed complex associations between plasma metabolites and comorbidities like gout and cardiac disorders. We could further validate those associations on test data.

Emerging datasets provide more and more layers of information, which makes data analysis increasingly complex. This high complexity leaves the researcher with an overwhelming amount of possible hypotheses requiring extensive tests and validations. Thus, there is an urgent need for automated tools that hint towards interesting observations. Here, MGMs are powerful analysis methods, because they condense the available data into graphs that researchers can easily read and interpret. Moreover, MGMs consider the whole system of variables and measurements simultaneously. As a consequence, they correct for confounding factors, such as age, gender, and medications, automatically. Thus, if the MGM observes an interesting association, it is likely not a bystander effect of other variables if those are part of the analysis itself. We showed this in a scenario where we corrected for artificially selected confounders. Particularly, we could demonstrate that confounder adjustment can be necessary to reveal associations, as exemplified for the association of gout with high alcohol consumption. Finally, we illustrated that the MGM discovered an association of TMAO with cardiac arrhythmia, which remains significant after adjustment for variables selected by expert knowledge. As described above, the association of TMAO with cardiac disorders has gained much attention in the recent literature. Here, we report associations between TMAO and cardiac arrhythmia and cardiac infarction revealed by an untargeted, hypothesis-free screening approach in a large-scale cohort of adult CKD stage 3 patients.

To thoroughly evaluate the estimation performance of our MGM algorithm, we used two independent approaches. We evaluated both the predictive performance of the identified first order neighborhoods on independent test data and we inspected the recovery of associations well-known in the literature. Such evaluation steps are important sanity checks to establish a novel method and they allow to assess the robustness of new associations, where the ground truth is unknown.

In general, other proposed multivariate data integration machine learning techniques, e.g., generalized linear models, naïve Bayes Classifiers, Random Forests, LASSO regression, etc.^31^, only focus on one specific hypothesis or outcome at a time, whereas our MGM approach investigates all possible associations between all variables simultaneously. In comparison to other probabilistic graphical models such as Gaussian Graphical Models, our approach facilitates the statistical evaluation of both continuous and discrete variables at the same time. The data integration approach relies on a well-defined probability density function describing all variable dependencies simultaneously. Just recently, the application of deep learning techniques such as Neural Networks on large-scale biomedical data emerged^31^. However, the interpretation as well as the representation of these models is not straightforward, and in general, they require even larger sample sizes than available in our study. In contrast, our trained MGM models can be easily visualized as networks, which offer a fast access to a large amount of extremely condensed association information.

However, we would also like to point out certain limitations. First, MGMs are undirected graphical models, which do not include information about causality. In the context of Gaussian graphical models, the estimation of causal relations from observational data was investigated by several authors^32–34^. Combining those methods with MGMs could further strengthen our data integration approach. Nevertheless, associations identified by the MGM approach can generate hypotheses, and causal relationships could then be further explored by additional experiments. Second, the estimation of an MGM requires a complete data matrix without any missing data. Consequently, we only included patients in our study with fully recorded clinical, demographic, drug information, and NMR data. Here, data imputation could further strengthen this approach. Third, our current data integration method does not cover longitudinal data measured across several time-points. The inclusion of, e.g., information about patient survival taking censoring into account would be an important extension of our approach. Fourth, like any statistical data analysis method, our MGM approach is sample-size dependent. Especially, in cases like the one described here where the number of estimated parameters (388521 different edge weights) is substantially larger than the number of samples (3705) it is important to control overfitting to reduce the number of false positive associations. This step is not unique and several strategies can be applied, e.g., taking the *l*_1_ (LASSO) or *l*_2_ (ridge) norm together with different weighting schemes. Note that these strategies particularly remove weak associations. Fifth, our MGM approach is currently restricted to linear relationships among variables and does not consider possible higher-order interactions. Haslbeck and Waldorp, e.g., proposed node-wise regression algorithms to estimate higher-order MGMs^35^, and the extension of our MGM approach to higher-order interactions would further generalize our workflow. Finally, the dependence of our data integration method on *a priori* chosen data preprocessing methods has not been evaluated yet. Especially, in metabolomics studies of complex biofluids such as plasma or, even more pronounced, urine, both the performance with regard to association recovery and the identity of these associations can be heavily confounded by the applied normalization technique, as illustrated, e.g., by^36^ and^37^. In case of linear or logistic regression, *zero-sum* regression^38,39^ was proposed to overcome these limitations^37^, and the development of inherently normalization- or scaling-invariant MGMs might further strengthen our data integration approach.

Here, we used NMR spectroscopy to obtain metabolic information. One of the main advantages of NMR is its robustness^40,41^, which is especially important in larger studies comprising up to several thousand samples. However, in contrast to mass spectrometry NMR is generally less sensitive with higher limits of quantification in the low micromolar range. As a consequence, NMR signals of low abundant metabolites may contain a considerable amount of measurement noise, which also influences the estimated MGM. Note that in this context the above described penalization strategies to control overfitting are particularly powerful to minimize the number of false positive associations.

In summary, we tested MGMs for the integrative analysis of categorical and continuous variables in the context of CKD. We illustrated its application, proposed two independent strategies for model evaluation, provided software to estimate MGMs, and exemplified their interpretation. Finally, we reported novel associations between the plasma metabolite TMAO and cardiac arrhythmia as well as infarction in adult CKD stage 3 patients.

## Methods

### Cohort description

The study cohort comprises 3705 participants of the German Chronic Kidney Disease (GCKD) study, whose detailed baseline clinical and demographic characteristics are described elsewhere^11,12^. It was approved by the local ethics committees and registered in the national registry for clinical studies (DRKS 00003971). All study procedures and protocols were approved by the ethics committees of all participating institutions (Friedrich-Alexander-University Erlangen-Nuremberg, Medical Faculty of the Rheinisch-Westfälische Technische Hochschule Aachen, Charité—University Medicine Berlin, Medical Center—University of Freiburg, Medizinische Hochschule Hannover, Medical Faculty of the University of Heidelberg, Friedrich-Schiller-University Jena, Medical Faculty of the Ludwig-Maximilians-University Munich, Medical Faculty of the University of Würzburg). The study was carried out in accordance with relevant guidelines and regulations. Written declarations of informed consent had been obtained from all study participants before inclusion. From each patient, one EDTA-plasma specimen had been collected at the baseline time-point and stored at −80 °C until NMR measurement.

### Clinical and demographic variables

For this study, we included 17 clinical chemistry parameters measured by SYNLAB International GmbH (Munich, Germany) and Central Lab, University Hospital Erlangen, Germany^12^, 73 demographic parameters including age, sex, disease history and lifestyle factors, and 46 different drug treatments, resulting in a total of 136 clinical variables. Supplementary Table S1 provides a list of all included variables, their corresponding distributions, as well as assessment information.

### NMR spectroscopy

For NMR measurements, 400 *μ*L of unfiltered EDTA-plasma were mixed with 200 *μ*L of 0.1 mol/L phosphate buffer at pH 7.4, 50 *μ*L of 0.75% (w) 3-trimethylsilyl-2,2,3,3-tetradeuteropropionate (TSP) dissolved in deuterium oxide, and 10 *μ*L of 81.97 mmol/L formic acid (all from Sigma-Aldrich, Taufkirchen, Germany), the latter serving as internal standard for referencing and quantification^42^. NMR experiments were carried out on a 600 MHz Bruker Avance III (Bruker BioSpin GmbH, Rheinstetten, Germany) employing a triple-resonance (^1^H, ^13^C ^31^P, ^2^H lock) cryogenic probe equipped with z-gradients and an automatic cooled sample changer. More details are provided in the Supplementary Methods section 1.1.

NMR signals were assigned to known metabolites by comparison with reference spectra of pure compounds acquired under equal experimental conditions employing the Bruker Biofluid Reference Compound Database BBIOREFCODE 2-0-3^43^.

### Data preprocessing

All continuous clinical and demographic variables except for age, systolic and diastolic blood pressure were log_2_ transformed to remove heteroscedasticity. Recoding of discrete variables is detailed in Supplementary Table S1. In summary, we included 25 continuous and 111 discrete clinical and demographic variables, respectively.

All NMR spectra were referenced with respect to the formic acid signal at 8.463 ppm. Since signal positions between spectra may be subject to minor shifts due to slight differences in pH, salt concentration, and/or temperature between samples, equidistant binning was employed to compensate for these effects. More details can be found in the Supplementary Methods section 1.2.

For further analysis, data were imported into *R* version 3.2.1 (Development Core Team 2009). To minimize heteroscedasticity of the NMR data, bucket intensities were log_2_ transformed and additionally subjected to mean-value subtraction.

All continuous variables were scaled to standard units.

### Mixed graphical models probability density function

Integration of all discrete and continuous variables is achieved by estimating the conditional dependencies between them by a Mixed Graphical Model (MGM)^16^. MGMs are undirected probabilistic graphical models, where each node corresponds to one variable, and the edges between two nodes represent a conditional dependency between them given all other variables in the graphical model. If there exists no edge between two nodes, these two variables are conditionally independent of each other given all other variables in the MGM.

Lee and Hastie^44^ proposed to describe the joint probability $p(x,y;\Theta )$ as a pairwise graphical model:

1 $$\begin{array}{lll}p(x,y;\Theta ) & \propto  & \exp (\frac{1}{2}\,\mathop{\sum }\limits_{s\mathrm{=1}}^{p}\,\mathop{\sum }\limits_{t\mathrm{=1}}^{p}\,{\beta }_{st}{x}_{s}{x}_{t}+\mathop{\sum }\limits_{s\mathrm{=1}}^{p}\,{\alpha }_{s}{x}_{s}\\  &  & +\,\mathop{\sum }\limits_{s\mathrm{=1}}^{p}\,\mathop{\sum }\limits_{j\mathrm{=1}}^{q}\,{\rho }_{sj}({y}_{j}){x}_{s}+\frac{1}{2}\,\mathop{\sum }\limits_{j\mathrm{=1}}^{q}\,\mathop{\sum }\limits_{r\mathrm{=1}}^{q}\,{\varphi }_{rj}({y}_{r},{y}_{j})).\end{array}$$

Here, *x*_*s*_ denotes the *s*th of *p* continuous variables, *y*_*j*_ denotes the *j*th of *q* discrete variables with *L*_*j*_ states, *β*_*st*_ represents the continuous - continuous edge, and *α*_*s*_ the continuous node potential, respectively, ${\rho }_{sj}$ is the continuous - discrete edge potential, represented as a vector of size *L*_*j*_, ${\varphi }_{rj}$ is the discrete - discrete edge potential, and $\Theta =[\{{\beta }_{st}\},\{{\alpha }_{s}\},\{{\rho }_{sj}\},\{{\varphi }_{rj}\}]$ summarizes the whole parameter space. $\frac{1}{2}\,{\sum }_{s=1}^{p}\,{\sum }_{t=1}^{p}\,{\beta }_{st}{x}_{s}{x}_{t}+{\sum }_{s=1}^{p}\,{\alpha }_{s}{x}_{s}$ describes conditional dependencies between two continuous variables *x*_*s*_ and *x*_*t*_, corresponding to the probability density function of a Gaussian Graphical Model (GGM)^44^. If ${\beta }_{st}=0$, no edge between *x*_*s*_ and *x*_*t*_ appears in the MGM, indicating an independency between these two nodes conditioned against all other nodes in the graphical model. Analogously, ${\sum }_{j=1}^{q}\,{\sum }_{r=1}^{q}\,{\varphi }_{jr}({y}_{j},{y}_{r})$, which corresponds to a discrete pairwise Markov Random Field^44^, describes conditional dependencies between two discrete variables *y*_*j*_ and *y*_*r*_ with *L*_*j*_ and *L*_*r*_ states, respectively. If all entries of the matrix ${\varphi }_{jr}$ are equal to zero, the two discrete variables are conditionally independent given all others. Finally, ${\sum }_{s=1}^{p}\,{\sum }_{j=1}^{q}\,{\rho }_{sj}({y}_{j}){x}_{s}$ gives the conditional dependencies between a continuous variable *x*_*s*_ and a discrete variable *y*_*j*_, represented by a vector of size *L*_*j*_. If all entries of this vector are equal to zero, the two variables are conditionally independent given all other variables in the MGM. In summary, if the whole parameter space $\Theta =[\{{\beta }_{st}\},\{{\alpha }_{s}\},\{{\rho }_{sj}\},\{{\varphi }_{rj}\}]$ is determined, we are able to fully describe all conditional dependencies between all variables in the considered system, here the population under investigation in the GCKD study, which can be represented in a network. The parameter estimation was carried out employing a pseudo-likelihood method as detailed in the Supplementary Methods section 1.3.

## Supplementary information

MGM_suppl_26_07_2019_merged_2

Supplementary_File_2_resubmission

## Data Availability

NMR spectra are available via the publicly accessible MetaboLights database https://www.ebi.ac.uk/metabolights/ accession ID MTBLS798. Patients provided written informed consent for their data to be shared within the scope of scientific collaborations. The authors should therefore be contacted with collaboration requests.

## Code Availability

The MGM implementation is available in Supplementary File 2.
