# Supplementary material for: A multi-source data integration approach reveals novel associations between metabolites and renal outcomes in the German Chronic Kidney Disease study
Source: Sci Rep. 2019 Sep 27;9:13954. doi: 10.1038/s41598-019-50346-2 (PMC6764972; doi:10.1038/s41598-019-50346-2)
Supplement: Supplementary file 1 — MGM_suppl_26_07_2019_merged_2 [file 41598_2019_50346_MOESM1_ESM.pdf]

Supplementary information for: A multi-source data integration approach reveals novel associations between metabolites and renal outcomes in the German Chronic Kidney Disease study

Michael Altenbuchinger[1,*], Helena U. Zacharias[2,*], Stefan Solbrig[3], Andreas Schäfer[3], Mustafa Büyüközkan[2], Ulla T. Schultheiß[4,5], Fruzsina Kotsis[4], Anna Köttgen[4], Rainer Spang[1], Peter J. Oefner[6], Jan Krumsiek[2,7], and Wolfram Gronwald[6, †]

[1]Statistical Bioinformatics, Institute of Functional Genomics, University of Regensburg, Am Biopark 9, 93053 Regensburg, Germany.
[2]Institute of Computational Biology, Helmholtz Zentrum München, Ingolstädter Landstraße 1, 85764 Neuherberg, Germany.
[3]Department of Physics, University of Regensburg, Universitätsstraße 31, 93053 Regensburg, Germany.
[4]Institute of Genetic Epidemiology, Department of Biometry, Epidemiology, and Medical Bioinformatics, Faculty of Medicine and Medical Center, University of Freiburg, 79106 Freiburg, Germany.
[5]Department of Nephrology, Medical Center, University of Freiburg, 79106 Freiburg, Germany.
[6]Institute of Functional Genomics, University of Regensburg, Am Biopark 9, 93053 Regensburg, Germany.
[7]Institute of Computational Biomedicine, Weill Cornell University, New York, NY 10021, USA.
[*]These authors contributed equally to this work.
[†]Correspondence: Wolfram.Gronwald@klinik.uni-regensburg.de, Tel.: +49-941-943-5015

List of Supplementary Files

Supplementary File 1	Supplementary Methods, Results, and Figures.
Supplementary File 2	Exemplary Mixed Graphical Model algorithm Python-code.
Supplementary Table 1	Information on clinical and demographic variables.

1 Supplementary Methods

1.1 NMR spectroscopy: measurement details

Since the presence of broad protein signals might hinder the interpretation of metabolite signals, 1D ^{1}H spectra employing a Carr-Purcell-Meiboom-Gill (CPMG) pulse-sequence for the suppression of macromolecular signals were acquired for each EDTA-plasma specimen. For each 1D ^{1}H CPMG spectrum, 128 scans were collected into 73728 data points employing presaturation during relaxation for water suppression. Four dummy scans were acquired prior to measurement, the spectral width was 20.02 ppm, the relaxation delay was 4 s and the acquisition time amounted to 3.07 s. The filtering delay amounted to 0.08 s, resulting in a total acquisition time of about 16 min per sample.

1.2 NMR data preprocessing

The spectral region from 9.5 - 0.5 ppm was evenly split into 900 bins of 0.01 ppm width using Amix VIEWER 3.9.13 (Bruker BioSpin GmbH, Rheinstetten, Germany). All spectral data were scaled relative to the formic acid signal from 8.5 - 8.45 ppm in order to reduce variations in spectrometer performance. NMR buckets corresponding to the remainders of the suppressed water signal as well as minor contributions from urea (6.0 - 4.5 ppm), formic acid (8.47 - 8.45 ppm), as well as dimethylamine (2.73 - 2.72 ppm) have been excluded prior to data analysis, resulting in a total number of 743 NMR buckets.

1.3 Pseudo-log-likelihood, LASSO penalty, and algorithmic implementation

A maximum likelihood estimate (MLE) of Eq. (1) in the main text requires a summation over all discrete states, which is numerically only feasible for small numbers of discrete variables. As an alternative, we use the pseudo-likelihood method of [1], where the negative pseudo log-likelihood is given by

$$\mathcal{L}(\Theta|x,y) = -\sum_{s=1}^{p} \log p(x_s|x_{\setminus s}, y;\Theta) - \sum_{r=1}^{q} \log p(y_r|x, y_{\setminus r};\Theta)\,. \tag{1}$$

Here, $p(x_s|x_{\setminus s}, y;\Theta)$ is the conditional distribution of a Gaussian variable x_s, and $p(y_r|y_{\setminus r,}, x;\Theta)$ is the conditional distribution of a discrete variable y_r with L_r states:

$$\begin{aligned} &p(x_s|x_{\setminus s}, y;\Theta) = \\ &\frac{\sqrt{-\beta_{ss}}}{\sqrt{2\pi}} \exp\left(\frac{\beta_{ss}}{2}\left(\frac{\alpha_s + \sum_j \rho_{sj}(y_j) + \sum_{t\neq s}\beta_{st}x_t}{\beta_{ss}} + x_s\right)^2\right), \\ &p(y_r|y_{\setminus r,}, x;\Theta) = \\ &\frac{\exp\left(\sum_s \rho_{sr}(y_r)x_s + \frac{1}{2}\phi_{rr}(y_r,y_r) + \sum_{j\neq r}\phi_{rj}(y_r,y_j)\right)}{\sum_{l=1}^{L_r}\exp\left(\sum_s \rho_{sr}(l)x_s + \frac{1}{2}\phi_{rr}(l,l) + \sum_{j\neq r}\phi_{rj}(l,y_j)\right)}\,. \end{aligned} \tag{2}$$

Note, that the pseudo-log-likelihood method does not require a summation over the whole state space, which makes it computationally more tractable than MLE.

We augmented the negative pseudo-log-likelihood of (1) by a LASSO penalty to calibrate overfitting

$$\lambda\left(\sum_{s=2}^{p}\sum_{t=1}^{s-1} w_{st}|\beta_{st}| + \sum_{s=1}^{p}\sum_{j=1}^{q} w_{sj}||\rho_{sj}||_1 + \sum_{j=2}^{q}\sum_{r=1}^{j-1} w_{rj}||\phi_{rj}||_1\right)\,, \tag{3}$$

where $||.||_1$ is defined as the element-wise matrix norm,

$$||\phi_{rj}||_1 = \sum_{l=1}^{L_r} \sum_{m=1}^{L_j} |\phi_{rj}(l,m)| \quad \text{and} \quad ||\rho_j||_1 = \sum_{l=1}^{L_j} |\rho_j(l)| \,. \tag{4}$$

The weights w_{st}, w_{sj}, and w_{rj} were chosen as

$$w_{st} = \sigma_s \sigma_t \,, \quad w_{sj} = \sigma_s \sqrt{\sum_b q_b(1-q_b)} \,,$$
$$w_{rj} = \sqrt{\sum_a p_a(1-p_a) \sum_b q_b(1-q_b)} \,, \tag{5}$$

where σ_s is the standard deviation of the continuous variable x_s and $p_a = Pr(y_r = a)$ and $q_b = Pr(y_j = b)$ [1]. For the multinomial variables, our parameter space is redundant [1]. This was taken into account by multiplying the penalty factors which correspond to baseline levels by a factor of 10. This forces their couplings to zero.

Optimization problem Eq. (3) is convex [1] and can be efficiently solved by proximal algorithms. We implemented our software in Python, where we used NumPy [2], and the Python package *apgpy* accessed under `https://github.com/bodono/apgpy`, which performs the proximal gradient descent with Nesterov's acceleration and adaptive restarts [3].

1.4 Calibration of the penalty parameter λ

We chose the penalty parameter λ according to the Extended Bayesian Information Criterion [4, 5]

$$\text{EBIC}_\gamma(\mathbf{E}) = -2l_n(\hat{\Theta}(\mathbf{E})) + |\mathbf{E}|\log n + 4|\mathbf{E}|\gamma \log P, \tag{6}$$

where γ is a parameter that can be chosen in the interval $[0,1]$, n is the number of measurements and P is the number of variables. $\mathbf{E}$ is the set of edge parameters unequal to zero, and for $l_n(\hat{\Theta}(\mathbf{E}))$ we used the corresponding maximized pseudo-log-likelihood. For calculating both P and $\mathbf{E}$ we removed the baseline levels from the adjacency matrix. The minimum of Eq. (6) determines the penalty parameter λ, which we estimated by line search. This line search sweeps the λ-sequence $\lambda_0 \cdot 2^{2,1.75,1.5,1.25,\ldots,-4.5,-4.75,-5}$, with $\lambda_0 = \sqrt{\frac{\log(p+q)}{n}}$. Throughout the article, we discuss the model $\gamma = 1$, which corresponds to the sparsest model according to EBIC. For visualization, we used the *R* packages *qgraph* [6] and *igraph* [7], where we transformed the edge weights by the formula $f(x) = \log(1 + 10x)$.

1.5 Association analyses

Analyses were performed using the statistical analysis software *R* version 3.3.3. For logistic regression, we used the *R* package *logistf* [8].

2 Supplementary Results

2.1 Urinary albumin-to-creatinine ratio

The urinary albumin-to-creatinine ratio (*UACR*) is, besides the glomerular filtration rate (GFR), another important marker of renal function. Supplementary Figure 1a visualizes the first order neighborhood of *UACR*. The MGM trained on the training set connects *UACR* with urinary albumin (*ua*) by a strong positive edge, and with urinary creatinine (*ucrea*) by a strong negative edge, respectively. This correctly reflects the definition of *UACR*, $\log_2(UACR) = \log_2(ua/ucrea) = \log_2(ua) - \log_2(ucrea)$.

A third positive edge appears between *ua* and *ucrea*. This edge can be explained within the conditional dependence framework: two variables are conditionally dependent on each other, if both variables simultaneously change upon holding all other variables fixed. Let the value of *UACR* be fixed and change the value of *ua*. In order to fulfill the requirement that the *UACR* stays fixed, urinary creatinine has

to change accordingly to compensate the changes of urinary albumin and, thus, a positive edge appears between *ua* and *ucrea*.

We further observe positive edges between severe proteinuria (*proteinuria* > *300mg/dL*) and *ua* as well as $UACR$, respectively, correctly stating that patients with high urinary levels of albumin and accordingly high $UACR$ values are diagnosed with proteinuria. Correspondingly, absent or mild proteinuria (*proteinuria* < *30mg/dL*) is negatively associated with urinary albumin and $UACR$. Here, intermediate proteinuria (*30mg/dL* < *proteinuria* < *300mg/dL*) was considered as the baseline.

As shown in Supplementary Figure 1b, the identified neighborhood is almost perfectly predictive of $UACR$ on independent test data with a correlation coefficient between true and predicted $UACR$ values of $corr \sim 1$.

2.2 Alcohol consumption

Alcohol consumption in the GCKD cohort was assessed with a questionnaire about weekly alcohol intake. Study participants, who had stated to consume alcohol not more than twice a week were classified as "little or normal alcohol consumption", here treated as the baseline level, and all other study participants were classified as "high alcohol consumption". Supplementary Figure 2a displays the first order neighborhood of "high alcohol consumption". Interestingly, high alcohol consumption is strongly positively associated with male gender, acute renal failure ($acute\_fail$), high density lipoprotein (*hdl*), an NMR bucket at 1.145 ppm, containing broad lipid signals mainly identified as low density lipoprotein (LDL), two unidentified NMR buckets at 0.835 and 2.755 ppm, and an NMR bucket at 2.675 ppm, identified as citric acid (negatively correlated). Further, it is positively associated with an NMR bucket at 1.925 ppm, identified as acetate (major signal) and minor contributions from arginine and lysine, and an NMR bucket at 3.275 ppm, identified as trimethylamine-N-oxide (TMAO) and minor contributions from D-glucose and betaine.

Levels of large and medium-sized HDL as well as large LDL particles have been reported to be elevated by alcohol consumption [9, 10]. It is also reported that alcohol consumption is generally higher in men than in women [10]. Citric acid has been reported to negatively correlate with alcohol consumption in other studies [11].

As shown in Supplementary Figure 2b, the first order neighborhood of high alcohol consumption is predictive on independent test data with an AUC of 0.796.

3 Supplementary Figures

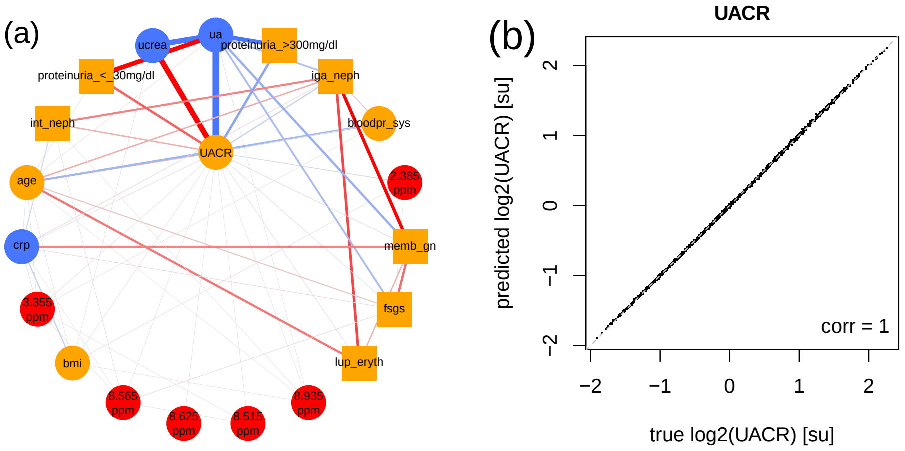

Figure 1: (a) First order neighborhood of urinary albumin-to-creatinine-ratio ($UACR$). $UACR$ is positively associated with urinary albumin (ua) (edge weight = 22.85) and negatively associated with urinary creatinine ($ucrea$) (edge weight = -7.19), respectively. The positive edge between ua and $ucrea$ (edge weight = 7.31) can be explained within the conditional dependence framework (see text). Two levels of proteinuria are connected to both $UACR$ and ua, respectively. High level of protein in the patient's urine ($proteinuria > 300mg/dL$) is positively associated, whereas proteinuria of less than 30mg/dL ($proteinuria < 30mg/dL$) is negatively associated with $UACR$ (edge weight = 0.57 and edge weight = -0.56, respectively) and ua (edge weight = 2.87 and edge weight = -3.53, respectively), respectively. Moderate proteinuria ($30mg/dL < proteinuria < 300mg/dL$) was considered as the baseline. (b) The diagram shows the predictions of $UACR$ on the y-axis (in standard units [su]) based on the neighbors of $UACR$ on independent test data compared to the true values plotted on the x-axis.

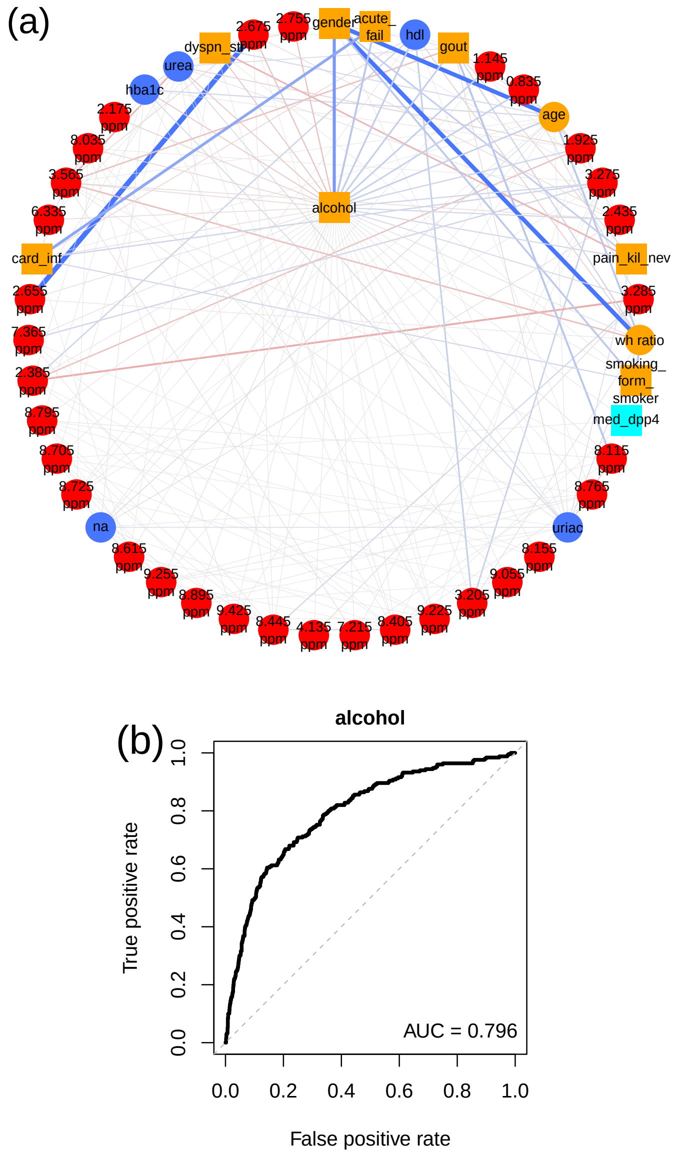

Figure 2: (a) First order neighborhood of high alcohol consumption (*alcohol*). Strong associations are observed between high alcohol consumption and male gender (edge weight = 0.68), acute kidney failure (*acute_fail*) (edge weight = 0.27), high density lipoprotein (*hdl*) values (edge weight = 0.21), gout (edge weight = 0.20), an NMR bucket at 1.145 ppm (edge weight = 0.19), containing broad lipid signals mainly identified as low density lipoprotein (LDL), an unidentified NMR bucket at 0.835 ppm (edge weight = 0.18), age (edge weight = 0.18), an unidentified NMR bucket at 2.755 ppm (edge weight = -0.14), an NMR bucket at 2.675 ppm (edge weight = -0.13), identified as citric acid, an NMR bucket at 1.925 ppm (edge weight = 0.13), identified as acetate (major signal) and minor contributions from arginine and lysine, and an NMR bucket at 3.275 ppm (edge weight = 0.13), identified as trimethylamine-N-oxide (TMAO) and minor contributions from D-glucose and betaine. (b) ROC curve for the neighborhood model of high alcohol consumption.

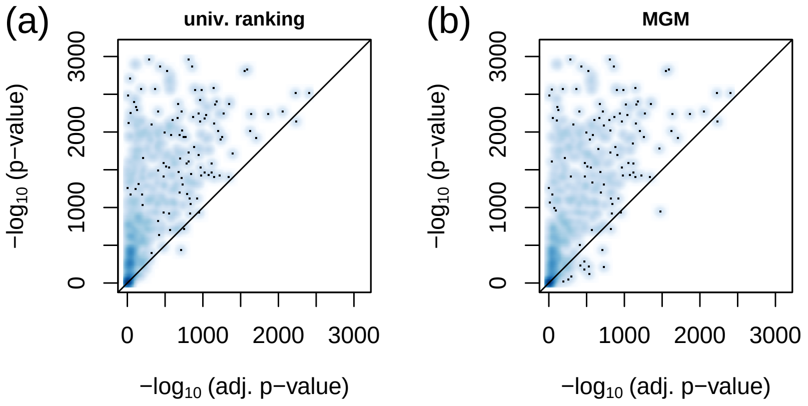

Figure 3: Smooth scatter plot of randomly adjusted p-values (x-axis) versus unadjusted (univariate) p-values (y-axis) for the univariate screening (a), and the MGM (b) in the training set.

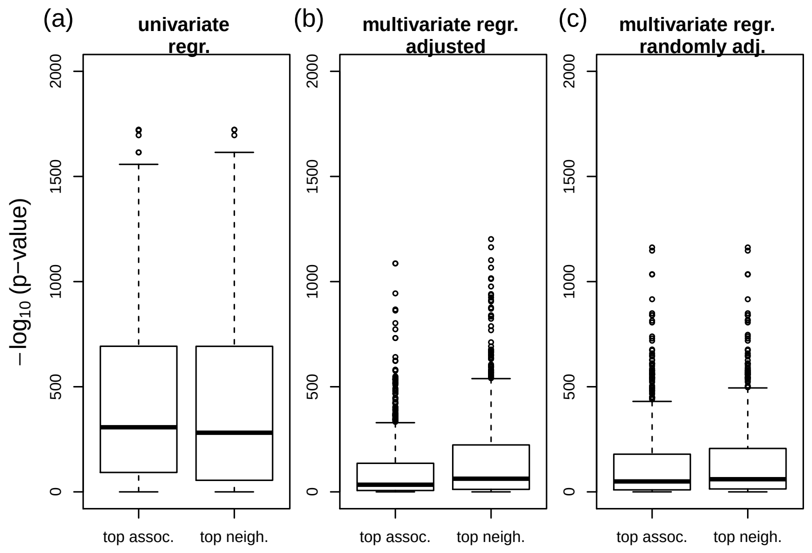

Figure 4: Effects of variable adjustment on univariate or MGM association analysis in the test set. (a) "top assoc." shows the distribution of $-\log_{10}(p\text{-values})$ derived from a univariate regression. Here, we calculated p-values between all possible pairs of variables and collected all top associations. (a) "top neigh." shows the analogous distribution, where the top feature was selected by largest absolute regression weight in the MGM neighborhood. (b) The corresponding plot, where the p-values were corrected by the top five confounder variables of the univariate and MGM screening, respectively. (c) The corresponding plot, where we adjusted for the same five randomly selected features for both methods.

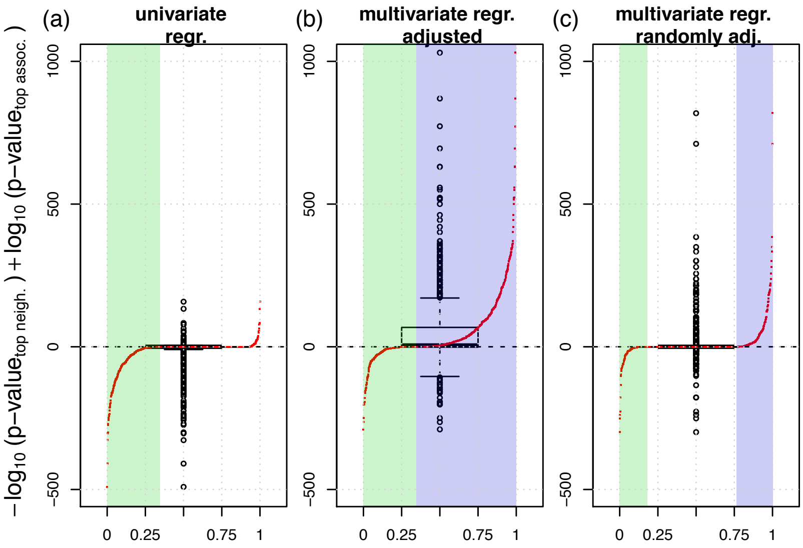

Figure 5: (a) to (c) show the differences between "top neigh." and "top assoc." in Figure 4a to c, respectively: (a) shows the $-\log_{10}(p\text{-values})$ of the MGM approach minus those of the univariate screening in Figure 4a, (b) shows the corresponding plot after adjusting for the respective top confounders, as shown in Figure 4b, and (c) shows the corresponding plot for adjusting for the randomly selected confounders, as shown in Figure 4c. The red points in each figure contrast the values on the y-axis with their respective rank. On the x-axis, the highest positive difference corresponds to 1 and the most negative to 0. The green shaded areas correspond to rank percentiles of negative, the violet shaded areas correspond to rank percentiles of positive differences.

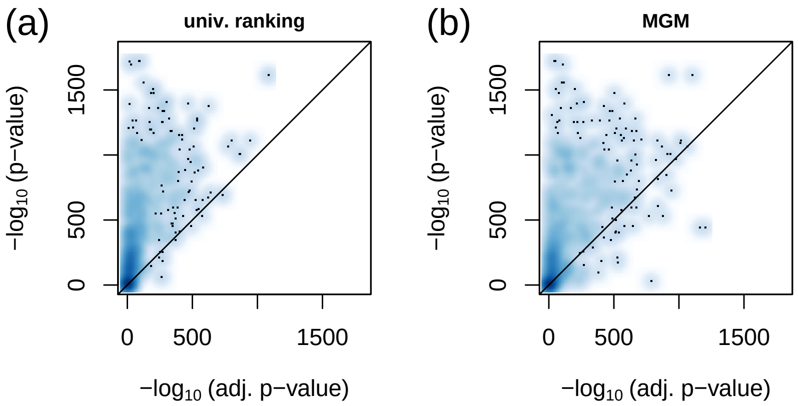

Figure 6: Smooth scatter plot of adjusted p-values (x-axis) versus unadjusted (univariate) p-values (y-axis) for the univariate screening (a), and the MGM (b) in the test set.

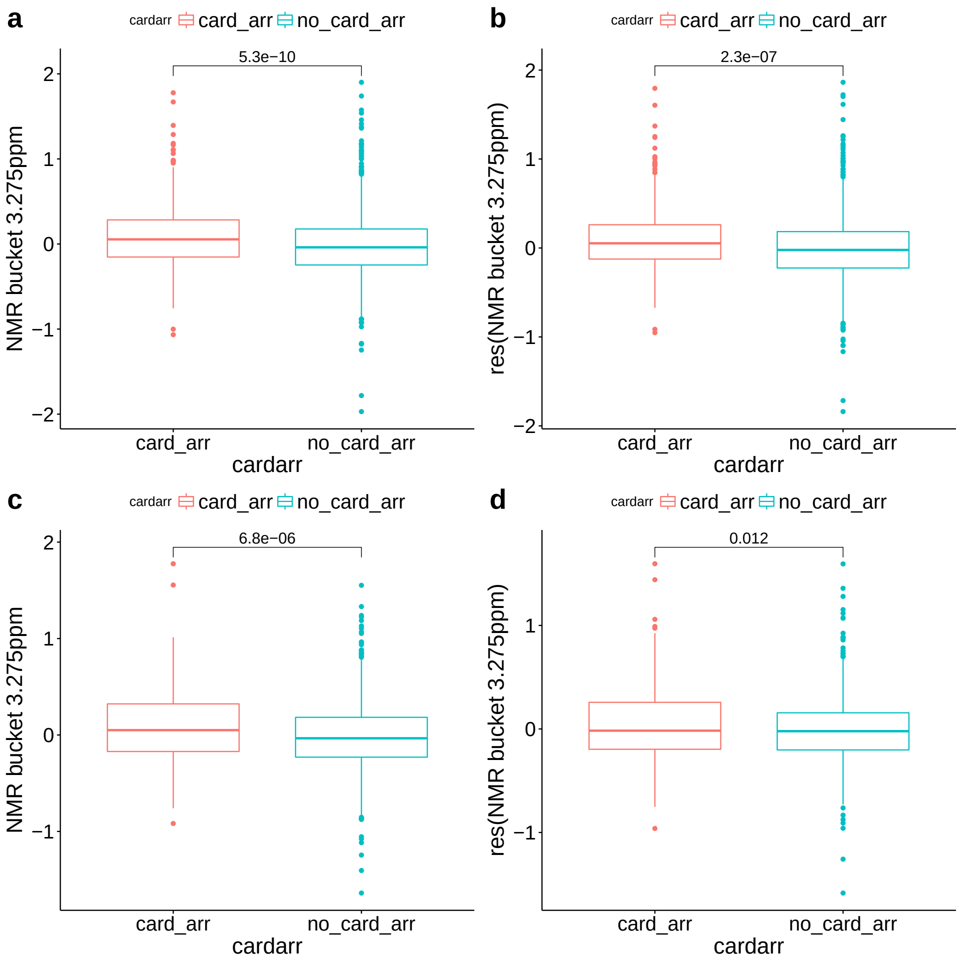

Figure 7: Boxplots comparing NMR signal intensities at 3.275ppm, which comprises NMR signals from trimethylamine-N-oxide (TMAO) (major), as well as minor signals from D-glucose and betaine. Log_2-transformed NMR signal intensities for patients with (*card_arr*) and without cardiac arrhythmia (*no_card_arr*) for both training (a) and test data (c), respectively, are shown. The residuals of $\log_2$-transformed NMR signal intensities after adjustment for BMI, systolic blood pressure, smoking status, and diabetes status for both training (b) and test data (d) are shown. P-values of corresponding t-tests are displayed above the boxes.

References

[1] Jason Lee and Trevor Hastie. Structure learning of mixed graphical models. In Carlos M. Carvalho and Pradeep Ravikumar, editors, Proceedings of the Sixteenth International Conference on Artificial Intelligence and Statistics, volume 31 of Proceedings of Machine Learning Research, pages 388–396, Scottsdale, Arizona, USA, 29 Apr–01 May 2013. PMLR.

[2] Stefan Van Der Walt, S Chris Colbert and Gail Varoquaoux. The NumPy array: a structure for efficient numerical computation. Computing in Science & Engineering, 13(2):22, 2011.

[3] Brendan O'Donoghue and Emmanuel Candes. Adaptive restart for accelerated gradient schemes. Foundations of computational mathematics, 15(3):715–732, 2015.

[4] Jiahua Chen and Zehua Chen. Extended bayesian information criteria for model selection with large model spaces. Biometrika, 95(3):759–771, 2008.

[5] Rina Foygel and Mathias Drton. Extended bayesian information criteria for gaussian graphical models. In Advances in neural information processing systems, pages 604–612, 2010.

[6] Sacha Epskamp, Angélique OJ Cramer, Lourens J Waldorp, Verena D Schmittmann, Denny Borsboom, et al. qgraph: Network visualizations of relationships in psychometric data. Journal of Statistical Software, 48(4):1–18, 2012.

[7] Gabor Csardi and Tamas Nepusz. The igraph software package for complex network research. InterJournal, Complex Systems:1695, 2006.

[8] Georg Heinze and Meinhard Ploner. logistf: Firth's Bias-Reduced Logistic Regression, 2016. R package version 1.22.

[9] Elizabeth R De Oliveira e Silva, MonnieMcGee Harper, Cynthia E Seidman, Jonathan D Smith, Jan L Breslow, Eliot A Brinton, et al. Alcohol consumption raises hdl cholesterol levels by increasing the transport rate of apolipoproteins ai and a-ii. Circulation, 2000.

[10] Kenneth J Mukamal, Rachel H Mackey, Lewis H Kuller, Russell P Tracy, Richard A Kronmal, Murray A Mittleman, and David S Siscovick. Alcohol consumption and lipoprotein subclasses in older adults. The Journal of Clinical Endocrinology & Metabolism, 92(7):2559–2566, 2007.

[11] Peter Würtz, Sarah Cook, Qin Wang, Mika Tiainen, Tuulia Tynkkynen, Antti J Kangas, Pasi Soininen, Jaana Laitinen, Jorma Viikari, Mika Kähönen, et al. Metabolic profiling of alcohol consumption in 9778 young adults. International journal of epidemiology, 45(5):1493–1506, 2016.

4 Supplementary Table 1

variable name	alias	assessment	type	category	mean +/- sd	unit	# per category	transformation
serum albumin	albu	measured by clinical chemistry at Synlab	continuous	clinical chemistry	38.38 +/- 4.25	g/l	-	log2-transformation
serum C-reactive protein	crp	measured by clinical chemistry at Synlab	continuous	clinical chemistry	4.50 +/- 7.74	mg/l	-	log2-transformation
serum cholesterol	chol	measured by clinical chemistry at Synlab	continuous	clinical chemistry	211.39 +/- 51.93	mg/dl	-	log2-transformation
serum high-density lipoprotein cholesterol	hdl	measured by clinical chemistry at Synlab	continuous	clinical chemistry	52.22 +/- 18.11	mg/dl	-	log2-transformation
serum low-density lipoprotein cholesterol	ldl	measured by clinical chemistry at Synlab	continuous	clinical chemistry	118.78 +/- 43.12	mg/dl	-	log2-transformation
serum triglycerides	trig	measured by clinical chemistry at Synlab	continuous	clinical chemistry	196.77 +/- 124.84	mg/dl	-	log2-transformation
serum creatinine	crea	measured by clinical chemistry at Synlab	continuous	clinical chemistry	1.51 +/- 0.47	mg/dl	-	log2-transformation
serum calcium	ca	measured by clinical chemistry at Synlab	continuous	clinical chemistry	2.27 +/- 0.15	mmol/l	-	log2-transformation
serum phosphate	pho	measured by clinical chemistry at Synlab	continuous	clinical chemistry	1.11 +/- 0.20	mmol/l	-	log2-transformation
serum cystatin C	cysC	measured by clinical chemistry at Synlab	continuous	clinical chemistry	1.51 +/- 0.48	mg/l	-	log2-transformation
serum urea	urea	measured by clinical chemistry at Synlab	continuous	clinical chemistry	28.46 +/- 12.20	mg/l	-	log2-transformation
serum uric acid	uriac	measured by clinical chemistry at Synlab	continuous	clinical chemistry	7.20 +/- 1.90	mg/dl	-	log2-transformation
serum sodium	na	measured by clinical chemistry at Synlab	continuous	clinical chemistry	139.68 +/- 3.07	mmol/l	-	log2-transformation

urinary albumin	ua	measured by clinical chemistry at Synlab	continuous	clinical chemistry	309.19 +/- 829.89	mg/l	-	log2-transformation
urinary creatinine	ucrea	measured by clinical chemistry at Synlab	continuous	clinical chemistry	81.82 +/- 55.32	mg/dl	-	log2-transformation
serum hemoglobin	hemo	measured by Central Lab, University Hospital Erlangen, Germany	continuous	clinical chemistry	13.61 +/- 1.66	g/dl	-	log2-transformation
glycated serum hemoglobin A1c	hba1c	measured by Central Lab, University Hospital Erlangen, Germany	continuous	clinical chemistry	45.59 +/- 11.15	mmol/mol	-	log2-transformation
patient age at baseline visit	age	assessed at baseline visit	continuous	demographic	59.87 +/- 12.0	years	-	-
estimated glomerular filtration rate by the CKD-EPI formula	eGFR	glomerular filtration rate estimated by CKD-EPI equation based on Synlab serum creatinine	continuous	demographic	49.56 +/- 18.14	ml/min per 1.73 m2	-	log2-transformation
urinary albumin to creatinine ratio	UACR	urinary albumin to creatinine ratio based on Synlab measurements	continuous	demographic	424.18 +/- 952.30	mg/g	-	log2-transformation
systolic blood pressure	bloodpr_sys	mean value of up to 3 systolic blood pressure values measured at baseline visit	continuous	demographic	139.46 +/- 20.15	mmHg	-	-
diastolic blood pressure	bloodpr_dias	mean value of up to 3 diastolic blood pressure values measured at baseline visit	continuous	demographic	79.49 +/- 11.73	mmHg	-	-
pulse	pulse	measured at baseline visit	continuous	demographic	70.59 +/- 12.11	bpm	-	log2-transformation

waist-hip ratio	wh ratio	quotient of measured waist-circumference to measured hip-circumference at baseline visit	continuous	demographic	0.94 +/- 0.09	-	-	log2-transformation
body mass index	bmi	measured body mass divided by measured squared body size assessed at baseline visit	continuous	demographic	29.59 +/- 5.83	kg/m2	-	log2-transformation
ever experienced cardiac infarction	card_inf	assessed at baseline visit	categorical	demographic	-	-	"yes": n=399; "no": n=3306	"no" was considered as baseline
ever underwent cardiac surgery	card_surg	assessed at baseline visit	categorical	demographic	-	-	"yes": n=335; "no": n=3370	"no" was considered as baseline
coronary angioplasty	cor_ves_enl	assessed at baseline visit	categorical	demographic	-	-	"yes": n=511; "no": n=3194	"no" was considered as baseline
ever diagnosed with heart failure	card_ins	assessed at baseline visit	categorical	demographic	-	-	"yes": n=656; "no": n=3049	"no" was considered as baseline
ever diagnosed with any cardiac arrhythmia	card_arr	assessed at baseline visit	categorical	demographic	-	-	"yes": n=725; "no": n=2980	"no" was considered as baseline
ever experienced stroke	stroke	assessed at baseline visit	categorical	demographic	-	-	"yes": n=279; "no": n=3426	"no" was considered as baseline
ever underwent carotid artery surgery	carot_surg	assessed at baseline visit	categorical	demographic	-	-	"yes": n=69; "no": n=3636	"no" was considered as baseline
ever underwent carotid artery angioplasty or stent placement	carot_enl	assessed at baseline visit	categorical	demographic	-	-	"yes": n=33; "no": n=3672	"no" was considered as baseline
catheter angiography of peripheral arteries	cont_ag	assessed at baseline visit	categorical	demographic	-	-	"yes": n=147; "no": n=3558	"no" was considered as baseline

including angioplasty of a peripheral artery								
ever underwent surgery to improve blood flow in legs	leg_surg	assessed at baseline visit	categorical	demographic	-	-	"yes": n=127; "no": n=3578	"no" was considered as baseline
diagnosed with aortic aneurysm	ao_aneu	assessed at baseline visit	categorical	demographic	-	-	"yes": n=91; "no": n=3614	"no" was considered as baseline
underwent retinal laser therapy due to diabetes	ret_las	assessed at baseline visit	categorical	demographic	-	-	"yes": n=279; "no": n=3426	"no" was considered as baseline
underwent amputation	amput	assessed at baseline visit	categorical	demographic	-	-	"yes": n=121; "no": n=3584	"no" was considered as baseline
ever diagnosed with high blood pressure	bl_pres	assessed at baseline visit	categorical	demographic	-	-	"yes": n=3303; "no": n=402	"no" was considered as baseline
ever diagnosed with elevated blood sugar levels	bl_sug	assessed at baseline visit	categorical	demographic	-	-	"yes": n=1362; "no": n=2243	"no" was considered as baseline
ever diagnosed with renal cancer	canc_ren	assessed at baseline visit	categorical	demographic	-	-	"yes": n=89; "no": n=3616	"no" was considered as baseline
ever diagnosed with breast cancer	canc_br	assessed at baseline visit	categorical	demographic	-	-	"yes": n=42; "no": n=3663	"no" was considered as baseline
ever diagnosed with cancer affecting female reproductive tract	canc_fem	assessed at baseline visit	categorical	demographic	-	-	"yes": n=45; "no": n=3660	"no" was considered as baseline
ever diagnosed with cancer affecting male reproductive tract	canc_mal	assessed at baseline visit	categorical	demographic	-	-	"yes": n=79; "no": n=3626	"no" was considered as baseline
ever diagnosed with any other malignoma	canc_oth	assessed at baseline visit	categorical	demographic	-	-	"yes": n=164; "no": n=3541	"no" was considered as baseline
ever diagnosed with asthma	asth	assessed at baseline visit	categorical	demographic	-	-	"yes": n=406; "no": n=3299	"no" was considered as

								baseline
ever diagnosed with chronic obstructive pulmonary disease	copd	assessed at baseline visit	categorical	demographic	-	-	"yes": n=232; "no": n=3473	"no" was considered as baseline
ever diagnosed with a gout attack	gout	assessed at baseline visit	categorical	demographic	-	-	"yes": n=872; "no": n=2833	"no" was considered as baseline
intake of prescription medication	drug	assessed at baseline visit	categorical	demographic	-	-	"yes": n=3666; "no": n=39	"no" was considered as baseline
intake of over the counter medication	hom_drug	assessed at baseline visit	categorical	demographic	-	-	"yes": n=1414; "no": n=2291	"no" was considered as baseline
intake of pain killers	pain_kil	assessed at baseline visit	categorical	demographic	-	-	"never": n=1535; "sometimes":n=1864; "regularly":n=306	"regularly" was considered as baseline
ever underwent temporary dialysis	temp_dial	assessed at baseline visit	categorical	demographic	-	-	"yes": n=165; "no": n=3540	"no" was considered as baseline
experienced angina pectoris	br_pain	assessed at baseline visit	categorical	demographic	-	-	"yes": n=1235; "no": n=2470	"no" was considered as baseline
ever experienced nocturnal dyspnea	dyspn	assessed at baseline visit	categorical	demographic	-	-	"yes": n=454; "no": n=3251	"no" was considered as baseline
use of extra pillows at night due to dyspnea	dyspn_cus	assessed at baseline visit	categorical	demographic	-	-	"yes": n=455; "no": n=3250	"no" was considered as baseline
ever experienced dyspnea during physical strain	dyspn_str	assessed at baseline visit	categorical	demographic	-	-	"yes": n=1494; "no": n=2211	"no" was considered as baseline
regular coughing with sputum	dyspn_cough	assessed at baseline visit	categorical	demographic	-	-	"yes": n=534; "no": n=3171	"no" was considered as baseline
renal disease due to type-1 diabetes mellitus	dm_typ1	telephone interview with treating physician	categorical	demographic	-	-	"yes": n=78; "no": n=3627	"no" was considered as baseline
renal disease due to type-2 diabetes	dm_typ2	telephone interview with	categorical	demographic	-	-	"yes": n=898; "no": n=2807	"no" was considered as

mellitus		treating physician						baseline
renal disease due to renal arterial stenosis	ren_artsten	telephone interview with treating physician	categorical	demographic	-	-	"yes": n=77; "no": n=3628	"no" was considered as baseline
renal disease due to Wegener's granulomatosis	morb_weg	telephone interview with treating physician	categorical	demographic	-	-	"yes": n=89; "no": n=3616	"no" was considered as baseline
renal disease due to microscopic polyangiitis	mic_polyang	telephone interview with treating physician	categorical	demographic	-	-	"yes": n=43; "no": n=3662	"no" was considered as baseline
renal disease due to systemic lupus erythematodes	lup_eryth	telephone interview with treating physician	categorical	demographic	-	-	"yes": n=95; "no": n=3610	"no" was considered as baseline
renal disease due to IgA-nephropathy	iga_neph	telephone interview with treating physician	categorical	demographic	-	-	"yes": n=336; "no": n=3369	"no" was considered as baseline
renal disease due to focal segmental glomerulosclerosis	fsgs	telephone interview with treating physician	categorical	demographic	-	-	"yes": n=159; "no": n=3546	"no" was considered as baseline
renal disease due to minimal change glomerulopathy	min_chan	telephone interview with treating physician	categorical	demographic	-	-	"yes": n=48; "no": n=3657	"no" was considered as baseline
renal disease due to membranous glomerulonephritis	memb_gn	telephone interview with treating physician	categorical	demographic	-	-	"yes": n=122; "no": n=3583	"no" was considered as baseline
renal disease due to interstitial nephropathy	int_neph	telephone interview with treating physician	categorical	demographic	-	-	"yes": n=177; "no": n=3528	"no" was considered as baseline
renal disease due to analgesic nephropathy	an_neph	telephone interview with treating physician	categorical	demographic	-	-	"yes": n=109; "no": n=3596	"no" was considered as baseline
renal disease due to nephrectomy due to tumour	tum_nephr	telephone interview with treating physician	categorical	demographic	-	-	"yes": n=104; "no": n=3601	"no" was considered as baseline
renal disease due to nephrectomy due to other reasons	oth_nephr	telephone interview with treating physician	categorical	demographic	-	-	"yes": n=70; "no": n=3635	"no" was considered as baseline
renal disease due to autosomal	adpkd	telephone interview with	categorical	demographic	-	-	"yes": n=151; "no": n=3554	"no" was considered as

dominant polycystic kidney disease		treating physician						baseline
renal disease due to kidney stones	kid_stones	telephone interview with treating physician	categorical	demographic	-	-	"yes": n=105; "no": n=3600	"no" was considered as baseline
renal disease due to recurrent inflammation	rec_inf	telephone interview with treating physician	categorical	demographic	-	-	"yes": n=84; "no": n=3621	"no" was considered as baseline
renal disease due to vesicoureteral reflux	ves_refl	telephone interview with treating physician	categorical	demographic	-	-	"yes": n=43; "no": n=3662	"no" was considered as baseline
renal disease due to vascular nephropathy	vasc_neph	telephone interview with treating physician	categorical	demographic	-	-	"yes": n=1519; "no": n=2186	"no" was considered as baseline
diagnosed with aortic valve stenosis	ao_sten	telephone interview with treating physician	categorical	demographic	-	-	"yes": n=53; "no": n=3652	"no" was considered as baseline
diagnosed with aortic valve insufficiency	ao_ins	telephone interview with treating physician	categorical	demographic	-	-	"yes": n=102; "no": n=3603	"no" was considered as baseline
diagnosed with mitral valve insufficiency	mit_ins	telephone interview with treating physician	categorical	demographic	-	-	"yes": n=182; "no": n=3523	"no" was considered as baseline
diagnosed with other heart valve anomalies	oth_ins	telephone interview with treating physician	categorical	demographic	-	-	"yes": n=128; "no": n=3577	"no" was considered as baseline
receives double renin-angiotensin system blockage medication	med_do_ras	aggregated baseline medication	categorical	drug treatment	-	-	"yes": n=290; "no": n=3415	"no" was considered as baseline
receives single renin-angiotensin system blockage medication	med_si_ras	aggregated baseline medication	categorical	drug treatment	-	-	"yes": n=2630; "no": n=1075	"no" was considered as baseline
receives blood-pressure altering medication	med_bp	aggregated baseline medication	categorical	drug treatment	-	-	"yes": n=3406; "no": n=299	"no" was considered as baseline
receives angiotensin	med_bp_ace	aggregated baseline	categorical	drug treatment	-	-	"yes": n=1750; "no": n=1955	"no" was considered as

converting enzyme inhibitor		medication						baseline
receives angiotensin II receptor blockers (arb)	med_bp_arb	aggregated baseline medication	categorical	drug treatment	-	-	"yes": n=1460; "no": n=2245	"no" was considered as baseline
receives diuretics	med_diuret	aggregated baseline medication	categorical	drug treatment	-	-	"yes": n=2183; "no": n=1522	"no" was considered as baseline
receives potassium-sparing diuretics	med_diuret_pot_sp	aggregated baseline medication	categorical	drug treatment	-	-	"yes": n=340; "no": n=3365	"no" was considered as baseline
receives thiazide diuretics	med_diuret_thiazid	aggregated baseline medication	categorical	drug treatment	-	-	"yes": n=1105; "no": n=2600	"no" was considered as baseline
receives aldosteron antagonists	med_diuret_aldoanta	aggregated baseline medication	categorical	drug treatment	-	-	"yes": n=287; "no": n=3418	"no" was considered as baseline
receives loop diuretics	med_diuret_loop	aggregated baseline medication	categorical	drug treatment	-	-	"yes": n=1381; "no": n=2324	"no" was considered as baseline
receives calcium channel blockers controlling blood pressure	med_bpcalciumanta	aggregated baseline medication	categorical	drug treatment	-	-	"yes": n=1349; "no": n=2356	"no" was considered as baseline
receives beta blockers controlling blood pressure	med_bpbetablock	aggregated baseline medication	categorical	drug treatment	-	-	"yes": n=2001; "no": n=1704	"no" was considered as baseline
receives central alpha receptor blockers	med_alpha_central	aggregated baseline medication	categorical	drug treatment	-	-	"yes": n=430; "no": n=3275	"no" was considered as baseline
receives pheripheral alpha receptor blockers	med_alpha_peripher	aggregated baseline medication	categorical	drug treatment	-	-	"yes": n=229; "no": n=3476	"no" was considered as baseline
receives drugs against diabetes mellitus	med_dm	aggregated baseline medication	categorical	drug treatment	-	-	"yes": n=995; "no": n=2710	"no" was considered as baseline
receives oral anti-diabetic drugs	med_dm_oral	aggregated baseline	categorical	drug treatment	-	-	"yes": n=540; "no": n=3165	"no" was considered as

		medication						baseline
receives insulin	med_dm_insulin	aggregated baseline medication	categorical	drug treatment	-	-	"yes": n=612; "no": n=3093	"no" was considered as baseline
receives metformin	med_metformin	aggregated baseline medication	categorical	drug treatment	-	-	"yes": n=241; "no": n=3464	"no" was considered as baseline
receives sulfonylureas	med_sulfonylhist	aggregated baseline medication	categorical	drug treatment	-	-	"yes": n=176; "no": n=3529	"no" was considered as baseline
receives glinides to treat type-2 diabetes mellitus	med_glinide	aggregated baseline medication	categorical	drug treatment	-	-	"yes": n=89; "no": n=3616	"no" was considered as baseline
receives dipeptidyl peptidase-4 (dpp4) drugs	med_dpp4	aggregated baseline medication	categorical	drug treatment	-	-	"yes": n=131; "no": n=3574	"no" was considered as baseline
receives drugs due to psychological disorders	med_psych1	aggregated baseline medication	categorical	drug treatment	-	-	"yes": n=462; "no": n=3243	"no" was considered as baseline
receives drugs due to psychological disorders in combination with analgetics	med_psych2	aggregated baseline medication	categorical	drug treatment	-	-	"yes": n=892; "no": n=2813	"no" was considered as baseline
receives antidepressants	med_antidepr	aggregated baseline medication	categorical	drug treatment	-	-	"yes": n=328; "no": n=3377	"no" was considered as baseline
receives analgetics	med_alg	aggregated baseline medication	categorical	drug treatment	-	-	"yes": n=579; "no": n=3126	"no" was considered as baseline
receives anti-dementia drugs	med_antidemenz	aggregated baseline medication	categorical	drug treatment	-	-	"yes": n=51; "no": n=3654	"no" was considered as baseline
receives lipid lowering drugs	med_lip_low	aggregated baseline medication	categorical	drug treatment	-	-	"yes": n=1867; "no": n=1838	"no" was considered as baseline
receives statins	med_statins	aggregated baseline medication	categorical	drug treatment	-	-	"yes": n=1737; "no": n=1968	"no" was considered as baseline
receives fibrates	med_fibrates	aggregated	categorical	drug treatment	-	-	"yes": n=68;	"no" was

		baseline medication					"no": n=3637	considered as baseline
receives cholesterol lowering drugs	med_chol_low	aggregated baseline medication	categorical	drug treatment	-	-	"yes": n=1763; "no": n=1942	"no" was considered as baseline
receives triglyceride lowering drugs	med_tg_low	aggregated baseline medication	categorical	drug treatment	-	-	"yes": n=172; "no": n=3533	"no" was considered as baseline
receives anti-anemic drugs	med_antianem	aggregated baseline medication	categorical	drug treatment	-	-	"yes": n=300; "no": n=3405	"no" was considered as baseline
receives iron-containing drugs	med_fer	aggregated baseline medication	categorical	drug treatment	-	-	"yes": n=160; "no": n=3545	"no" was considered as baseline
receives vitamin B 12	med_vit12	aggregated baseline medication	categorical	drug treatment	-	-	"yes": n=42; "no": n=3663	"no" was considered as baseline
receives folic acid	med_fol	aggregated baseline medication	categorical	drug treatment	-	-	"yes": n=69; "no": n=3636	"no" was considered as baseline
receives calcium	med_calc	aggregated baseline medication	categorical	drug treatment	-	-	"yes": n=440; "no": n=3265	"no" was considered as baseline
receives vitamin D	med_vitD	aggregated baseline medication	categorical	drug treatment	-	-	"yes": n=1126; "no": n=2579	"no" was considered as baseline
receives bisphosphonate	med_bisphosphonate	aggregated baseline medication	categorical	drug treatment	-	-	"yes": n=64; "no": n=3641	"no" was considered as baseline
receives anti-osteoporosis drugs	med_osteopor	aggregated baseline medication	categorical	drug treatment	-	-	"yes": n=73; "no": n=3632	"no" was considered as baseline
receives dual antiplatelet therapy	med_dual_antipl	aggregated baseline medication	categorical	drug treatment	-	-	"yes": n=73; "no": n=3632	"no" was considered as baseline
receives anti thrombotic drugs	med_antipl	aggregated baseline medication	categorical	drug treatment	-	-	"yes": n=1588; "no": n=2117	"no" was considered as baseline
receives vitamin K antagonists	med_vitK_ant	aggregated baseline	categorical	drug treatment	-	-	"yes": n=385; "no": n=3320	"no" was considered as

		medication						baseline
receives heparin	met_heparin	aggregated baseline medication	categorical	drug treatment	-	-	"yes": n=38; "no": n=3667	"no" was considered as baseline
receives antiplatelet therapy	med_antipl_agg	aggregated baseline medication	categorical	drug treatment	-	-	"yes": n=1235; "no": n=2470	"no" was considered as baseline
receives clopidogrel	med_clopido	aggregated baseline medication	categorical	drug treatment	-	-	"yes": n=141; "no": n=3564	"no" was considered as baseline
receives acetylsalicylic acid (asa)	med_asa	aggregated baseline medication	categorical	drug treatment	-	-	"yes": n=1198; "no": n=2507	"no" was considered as baseline
patient smoking status	smoking	self-reported smoking status	categorical	demographic	-	-	"former smoker": n=1603; "non-smoker": n=1502; "smoker": n=600	"non-smoker" was considered as baseline
patient proteinuria categories	proteinuria	aggregated baseline data	categorical	demographic	-	-	"<30mg/dl": n=1786; "30-300(incl.)mg/dl": n=851,">300mg/dl": n=1068	"30-300(incl.)mg/dl" was considered as baseline
patient gender	gender	aggregated baseline data	categorical	demographic	-	-	"male": n=2243; "female": n=1462	"female" was considered as baseline
patient alcohol intake	alcohol	self-reported weekly alcohol intake	categorical	demographic	-	-	"high alcohol consumption": n=722; "low/normal alcohol consumption": n=2983	"low/normal alcohol consumption" was considered as baseline
leading CKD disease cause diabetic nephropathy	diab_neph	aggregated baseline data, diagnosed if physician reported type-1 or type-2 diabetes mellitus or other diabetic nephropathy	categorical	demographic	-	-	"yes": n=980; "no": n=2725	"no" was considered as baseline
leading CKD	single_kidney	aggregated	categorical	demographic	-	-	"yes": n=225;	"no" was

disease cause single kidney		baseline data, diagnosed if treating physician reported tumour nephrectomy, living kidney donor, aggregated baseline data, nephrectomy due to other reasons, renal agenesis or other reason for single kidney					"no": n=3480	considered as baseline
leading CKD disease cause hypertensive nephropathy	hyp_neph	aggregated baseline data, diagnosed if physician reported renal artery stenosis, nephrosclerosis, renal infarction, or other vascular nephropathy	categorical	demographic	-	-	"yes": n=1519; "no": n=2186	"no" was considered as baseline
leading CKD disease cause acute renal failures	acute_fail	aggregated baseline data, diagnosed if physician reported post-ischemic, septic, or toxic acute renal failure	categorical	demographic	-	-	"yes": n=174; "no": n=3531	"no" was considered as baseline
positive disease history of diabetes mellitus	diabetic	aggregated baseline data, diagnosed if physician reported HbA1c >= 6.5% or patient receives at least one drug classified as ‘A10‘	categorical	demographic	-	-	"yes": n=1280; "no": n=2425	"no" was considered as baseline
positive disease	hypertension	aggregated	categorical	demographic	-	-	"yes": n=3558;	"no" was

history for hypertension		baseline data, diagnosed if patient has mean systolic blood pressure >= 140 mmHg, or mean diastolic blood pressure >= 90mmHg, or receives at least one drug classified as 'C02', 'C03', 'C07', 'C08', or 'C09'					"no": n=147	considered as baseline
