# Supplementary material for: A multi-source data integration approach reveals novel associations between metabolites and renal outcomes in the German Chronic Kidney Disease study
Source: Sci Rep. 2019 Sep 27;9:13954. doi: 10.1038/s41598-019-50346-2 (PMC6764972; doi:10.1038/s41598-019-50346-2)
Supplement: Supplementary file 2 — Supplementary_File_2_resubmission [file 41598_2019_50346_MOESM2_ESM.zip › Supplementary_File_2_resubmission/readme.html]

 


readme


# Supplementary Code

Our python3 implementation requires numpy, apgpy (https://github.com/bodono/apgpy), and matplotlib.

Run the example code (lambda=0) by calling "apply\_functions\_command\_line.py" as follows:

```
python3 apply_functions_command_line.py --iterations=10000 --oTol=1e-10 --X_file='X.npy' --D_file='D.npy' --lamseq_file='lam_seq0.npy' --levels_file='levels.npy' --results_folder='temp'
```

This estimates an MGM as described in the supplementary methods section. Here,

- --Iterations gives the maximum number of update steps of the gradient descent
- --oTol is a precision cutoff, corresponding to "eps" in apgpy
- --X\_file needs to be a \*.npy file that contains the data of the continuous variables (measurements in the rows and features in the columns)
- --D\_file needs to be a \*.npy file that contains the data of the categorical variables (measurements in the rows and features in the columns). Note that each level is encoded separately as 0 and 1.
- --lamseq\_file needs to be a \*.npy file that contains a sequence for the penalty parameter lambda (in decreasing order). Note, the first level of each discrete variable is chosen as the baseline. Thus, for lambda>0 the corresponding coefficients are forced to zero. See also supplementary methods.
- --levels\_file needs a \*.npy file with the levels of the categorical variables corresponding to the D\_file
- --results\_folder gives the folder for saving the results. Here, the matrices B correspond to the continuous-continuous couplings, Rho to the discrete-continuous couplings, and Phi to the discrete-discrete couplings. The file neglogli.npy contains the corresponding negative pseudo log-likelihoods normalized by the number of measurements (the constant term is neglected). The indices of B, Rho, and Phi correspond to the position in the provided lambda sequence.

For visualization run:

```
python3 make_example_plots.py
```

to visualize the results for lambda=0.

### Estimate MGMs for a lambda sequence

An example sequence is provided in 'lam\_seq.npy' (should be in a decreasing order).

```
python3 apply_functions_command_line.py --iterations=10000 --oTol=1e-10 --X_file='X.npy' --D_file='D.npy' --lamseq_file='lam_seq.npy' --levels_file='levels.npy' --results_folder='temp'
```
